# *N*-Heterocycles as Promising Antiviral Agents: A Comprehensive Overview

**DOI:** 10.3390/molecules29102232

**Published:** 2024-05-10

**Authors:** Gulraiz Ahmad, Maria Sohail, Muhammad Bilal, Nasir Rasool, Muhammad Usman Qamar, Codrut Ciurea, Luigi Geo Marceanu, Catalin Misarca

**Affiliations:** 1 Department of Chemistry, Government College University, Faisalabad 38000, Pakistan; gulchemist35@gmail.com (G.A.); mariasohail899@gmail.com (M.S.); 2 School of Chemistry and Chemical Engineering, Shandong University, Jinan 250100, China; muhammadbilalgcuf@gmail.com; 3 Institute of Microbiology, Faculty of Life Sciences, Government College University, Faisalabad 38000, Pakistan; musmanqamar@gcuf.edu.pk; 4 Division of Infectious Diseases, Geneva University Hospitals, 1205 Geneva, Switzerland; 5 Department of Microbiology and Molecular Medicine, University of Geneva, 1205 Geneva, Switzerland; 6 Faculty of Medicine, Transilvania University of Brasov, 500036 Brasov, Romania; marceanu@gmail.com (L.G.M.);

**Keywords:** viral diseases, *N*-heterocycles, antiviral activity, antiviral drugs

## Abstract

Viruses are a real threat to every organism at any stage of life leading to extensive infections and casualties. *N*-heterocycles can affect the viral life cycle at many points, including viral entrance into host cells, viral genome replication, and the production of novel viral species. Certain *N*-heterocycles can also stimulate the host’s immune system, producing antiviral cytokines and chemokines that can stop the reproduction of viruses. This review focused on recent five- or six-membered synthetic *N*-heterocyclic molecules showing antiviral activity through SAR analyses. The review will assist in identifying robust scaffolds that might be utilized to create effective antiviral drugs with either no or few side effects.

## 1. Introduction

Nitrogen, oxygen, and sulfur are the most prevalent heteroatoms incorporated in heterocyclic rings. Heterocyclic compounds represent a category of cyclic organic molecules, each featuring at least one heteroatom. Given their significant roles in numerous diseases, they hold a pivotal position among organic chemicals extensively employed across various biological disciplines [1,2]. More than 90% of new drugs contain heterocycles and the interface between chemistry and biology is crossed by heterocyclic compounds [3,4]. Experience has shown that compounds with biological activity are often derived from heterocyclic structures and, surprisingly, this structural class has received special attention [5]. The development of robust synthetic routes readily generates the bulk quantities of a desired compound and helps to accelerate the drug development process [6]. These ring structures, comprising heteroatoms, serve as fundamental frameworks in numerous biological compounds, including hemoglobin, DNA, RNA, vitamins, hormones, and more. Moreover, several medications approved by the FDA for the treatment of various ailments also incorporate these heterocyclic structures into their composition [7]. Furthermore, *N*-heterocyclic compounds exhibit a diverse spectrum of noteworthy biological properties. These encompass essential activities such as anti-HIV, antidiabetic, anticancer, insecticidal, anti-inflammatory, antibacterial, antioxidant, anticonvulsant, antiallergic, and enzyme inhibition effects. These assertions find ample support in the scientific literature [8,9,10,11,12,13,14,15,16]. 

Various disorders caused by viruses, affecting multiple body systems with respiratory infections, are a significant global health concern. Common viruses like adenovirus, paramyxovirus, orthomyxovirus, and picornaviruses (enteroviruses and rhinoviruses) contribute to respiratory illnesses like pharyngitis, pneumonitis, and influenza [17]. While various antiviral medications employ different mechanisms to combat viral infections, the need for novel treatments remains pressing due to factors like rapid viral spread, mutation, drug resistance, and gaps in treatment options for specific viral diseases. Current antiviral therapies face challenges, including the development of drug resistance, limited drug effectiveness within the body, and potentially severe side effects [18]. 

Antiviral drugs are usually targeted, and resistance often develops when they are used against the same infection or related viruses. It is crucial to develop new antiviral compounds with diverse mechanisms of action to address the issues associated with existing medications. The progress in antiviral drug development is influenced by ongoing research in medicinal chemistry. Currently, direct-acting antivirals (DAAs) used for viral infection treatment include Boceprevir, Telaprevir, BI-201335, TMC-435, Vaniprevir (MK-7009), RG-7227 (Danoprevir), ABT-450, ACH-1625, BIT-225, BMS-650032, GS-9256, and MK-5172. These are *N*-heterocyclic compounds functioning as virucidal antivirals. Apart from the cost of treatment, these drugs generally receive positive patient feedback [19,20,21]. Antiviral medications hinder viral replication by halting protein production, genome replication, or blocking host cell access. Initial antiviral drugs emerged in the 1960s. Vaccines have played a vital role in eradicating viruses like polio, rubella, and smallpox. However, current antiviral agents are not always fully effective due to viral mutations and may lead to side effects. Natural antiviral compounds offer advantages, such as easy degradation, low toxicity, specificity, environmental friendliness, and unique modes of action [22,23,24,25,26]. 

**Figure 1:**
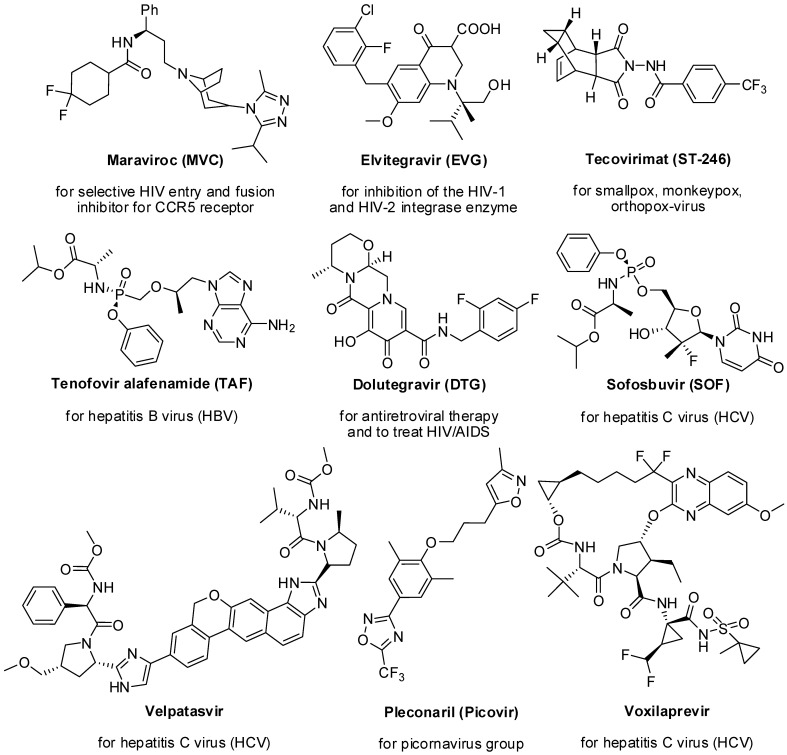
FDA-approved *N*-heterocyclic drugs.

The non-structural 5A protein (NS5A) plays a distinctive role in the replication of the hepatitis C virus (HCV) as an integral component of the HCV replication complex [27]. Recent introductions of oral NS5A inhibitors, such as Ledipasvir, Ombitasvir, Daclatasvir, Elbasvir, and Velpatasvir, have led to a significant reduction in viral load. This marks a revolutionary breakthrough in the treatment of hepatitis C [28,29]. Maximum current drugs are direct-acting antiviral compounds that target viral proteins, specifically. The existence of multidrug-resistant viruses is a major problem in present scientific research that should be measured as a priority for new methods of research [30,31]. Some of the FDA-approved *N*-heterocyclic antiviral drugs are listed in Figure 1.

This study has conducted a review of various *N*-heterocyclic compounds, including five- and six-ring systems that have been investigated for their antiviral properties. Some related research articles and reviews have been recently published for specific types of heterocycles, viruses, and small duration, e.g., Heterocycles as antiviral agents against SARS-CoV-2 [32], Investigation of antiviral *N*-Heterocycles as COVID-19 Drugs [33], *N*-heterocyclic compounds as antiviral agents in the last 5 years [34,35], Nitrogen/Sulfur Heterocyclic Compounds with Selective Antiviral Activities [36], and Common *N*-heterocycles (5 types) applied for antiviral purposes during the past ten years [37]. Compared to previously published articles, this comprehensive review highlights recent advancements in the exploration of twenty-two types of synthesized and natural nitrogen-containing heterocyclic compounds and nucleosides for their antiviral potential against all types of viruses, along with in-depth structure–activity relationship (SAR) assessments, during the last 15 years. This review will provide detailed knowledge of *N*-heterocycles as antiviral agents to the reader under one topic. First, five-membered *N*-heterocycles were discussed starting with pyrazole derivatives due to leading antiviral activity, and eleven other compounds (Imidazole, Thiazole, Thiazolidinone, Thiadiazole, Triazole, Oxazole/Oxadiazole, Pyrrole, Pyrrolidine, Indole, Isatin, and Indolizidine) were studied. Then, five- and six-membered fused *N*-heterocycles (Imidazo-Pyrimidine) were studied. After that, nine types of six-membered *N*-heterocycles (Pyrimidine, Triazine, Quinazoline/Quinazolin-ones, Pyrazine, Quinoxaline, Piperazine, Piperidine, Pyridine, and Quinolines) were discussed. In the end, miscellaneous *N*-heterocyclic derivatives with different moieties were studied. The key compounds identified in the study hold promise for the development of new antiviral drug candidates. This review article contributes to the identification of robust molecular frameworks that can be further developed into effective medications for the treatment of viral infections with minimal or no adverse effects.

## 2. Pyrazole Derivatives

Pyrazole molecules have been used as herbicides in agrochemicals and contain various medical and therapeutical properties, including ACE inhibitory, anti-inflammatory, anticancer, antimicrobial, and antiviral activities [38]. Pyrazole molecules are an essential component of a pharmacophoric scaffold with numerous pharmacological and biological applications. Most are accessible as antivirals for hepatitis A, HIV, HPAI (H5N1) influenza virus, HSV-1, hypoxia-inducible factor, and hepatitis C virus [39,40,41,42,43,44,45,46,47].

**Figure 2:**
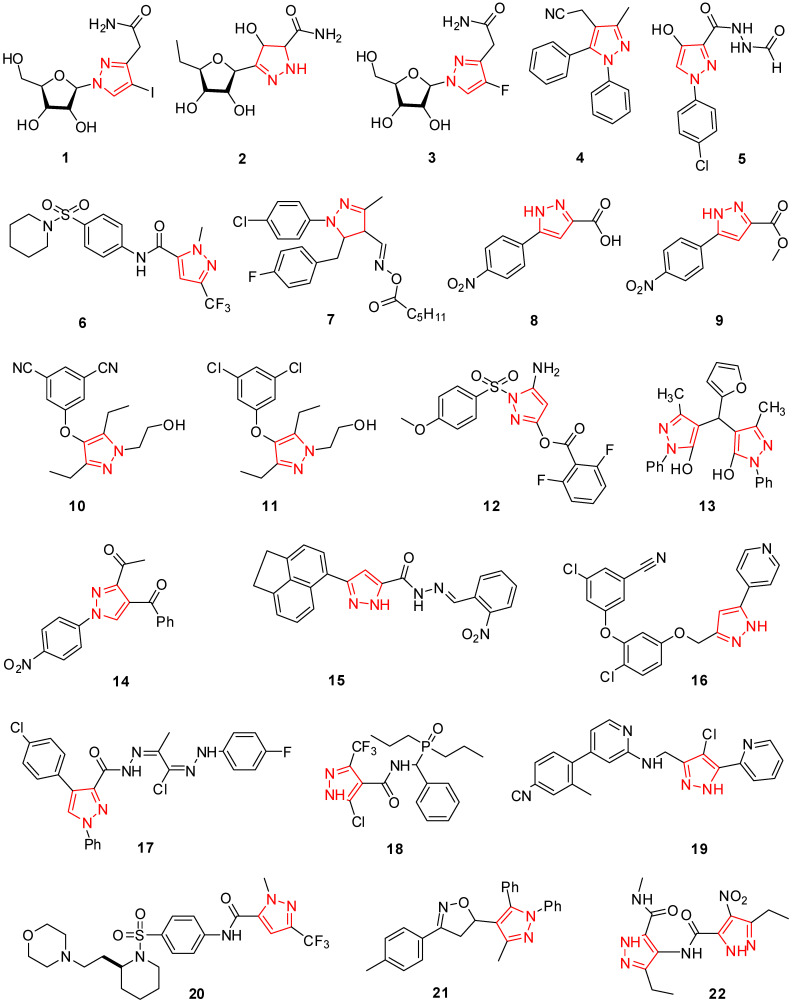
Pyrazole derivatives with antiviral activity.

Various pyrazolo[4,3-d]pyrimidine, pyrazolo[4,3]pyrimidine, and pyrazole ribonucleosides were evaluated for their in vitro antiviral activity against African swine fever virus (ASFV), herpes simplex virus type-1 (HSV-1), poliovirus, vesicular stomatitis virus (VSV), Coxsackievirus, and human immunodeficiency virus type-1 (HIV-1). Compound **1**, one of these pyrazole nucleosides (Figure 2), reduced the multiplication of HIV-1 in C8166 heavily infected cells [48]. Chen and Schneller created 5′-deoxypyrazofurin hybrids, which were investigated as antiviral agents against viruses of a wide range, including arena, toga, herpes, picon, myxo, pox, reo, rhabdoviruses, and retro. At concentrations ranging from 4 to 20 μg/mL, molecule **2** (Figure 2) showed notable activity towards the vaccinia virus in embryonic epidermis fibroblast cells, the influenza A virus in Madin-Darby canine kidney cells, the vesicular stomatitis virus in HeLa cells, and the respiratory syncytial virus in HeLa cells [49]. 

In vitro testing of a new fluoropyrazole ribonucleoside against the influenza virus was performed. Hybrid **3** had high efficacy towards influenza A and B, with I_50_ values of 0.2 and 0.4 μg/mL (Figure 2) [50]. A model class of 1,5-diphenylpyrazoles was invented by Genin et al. to prevent HIV-1, which functions as a non-nucleoside reverse transcriptase. Molecule **4** has strong efficacy against delavirdine-resistant P236L with an IC_50_ of 1.1 M and wild-type reverse transcriptase with an IC_50_ of 2.3 M (Figure 2) [51]. A new chain of 1-(4-chlorophenyl)-4-hydroxy-1*H*-pyrazole-3-carboxylic acid hydrazide derivatives were evaluated using reverse transcription polymerase chain reaction to see how they affected the development of the hepatitis C virus in the HepG2 hepatocellular carcinoma cell line. At 10–100 μg/mL, compound **5** (Figure 2) was shown to be efficient in blocking the proliferation of RNA HCV in both the (+) and (−) strands [52]. 

Model 1-methyl-3-(trifluoromethyl)-*N*-[4-pyrrolidinylsulfonyl)phenyl]-1*H*-pyrazole-5-carboxamide derivatives were tested against several measles virus (MV) genotypes and found to be effective inhibitors. When used in secondary viral titer reduction studies, the most efficient reagent **6** (Figure 2) exhibited its efficiency against live MV (0.012 ± 0.017 M) with no cytotoxicity [53]. The antiviral activity of a new series of ester compounds, such as pyrazolaldoxime, against TMV, was evaluated by Ouyang et al. The bioassay results revealed a range of poor to outstanding anti-TMV biocompatibility for these substances. For TMV CP, labeled molecule **7** showed greater affinity and improved biological activity (Figure 2) [45].

Some pyrazolo[3,4-d] pyrimidine and pyrazole derivatives were developed and evaluated for antiviral efficacy toward herpes simplex virus type-1 (HSV-1) and the hepatitis A virus (HAV) [43]. Zeng et al. developed new benzyl-substituted derivatives of 1*H*-pyrazole-3-carboxylic acid and readily observed the impacts on HIV replication and IN inhibition. The fantastic antiviral outcome was displayed by 5-(4-nitrophenyl) (4-nitrophenyl)-1*H*-pyrazole-3-carboxylic acid (**8**) and 3-(3-(benzyloxy)phenyl)isoxazole-5-carboxylic acid (**9**) with EC_50_ values of 253 and 3.6 μM, respectively (Figure 2) [54]. 

Mowbray et al. introduced new *N*-hydroxyethyl pyrazole compounds and tested in vivo anti-HIV efficacy. For several drug-resistant and wild-type HIV strains, molecule **10** (Figure 2) showed spectacular activity against the isolated RT enzymes [55]. Again, the same team developed the plan and manufactured a particular class of pyrazole-modified NNR-TIs, non-nucleoside HIV reverse transcriptase inhibitors. These substances maintain their effectiveness against clinically significant mutations and are effective against reverse transcriptase (RT) of the wild type. The 3,5-diethylpyrazole **11** compounds were discovered to be the series’ most active molecule (Figure 2) [56].

The synthesis of 3-substituted pyrazole derivatives of ester that were effective amphipathic stimulants of the NS2B-NS3 proteinase of the West Nile Virus was revealed by Siddique et al. Molecule **12** (Figure 2) was more significant than all the others, with an IC_50_ value of 1.96 μM [57]. In vitro*,* the antiviral efficacy of hybrids of 4,4′-(Arylmethylene)bis(1*H*-pyrazole-5-ol) developed by Sujatha et al. towards the peste des petits ruminant (PPRV) virus was evaluated. The most effective substance in the group, molecule **13**, had outstanding antiviral efficacy towards PPRV and outperformed the commonly prescribed drug Ribavirin (Figure 2) [58].

Riyadh et al. produced three novel pyrazole compounds and tested them for HCV antiviral efficacy. With an MIC value of 0.144 μg/mL, molecule **14** (Figure 2) from the series exhibited exceptional anti-HCV activity [46]. Shih et al. developed new pyrazole compounds with efficient and specific anti-influenza virus efficacy using a comparable cell-based neutralization experiment. We determined that compound **15** (BPR1P0034) has potent inhibitory action after testing 20,800 randomly chosen molecules from a collection (Figure 2). The significant findings indicate the value of developing innovative chemicals to combat influenza viruses [41].

In a range of new pyrazole compounds, Su et al. discovered powerful HIV-1 reverse transcriptase (RT) inhibitors, with practical antiviral ability in diseased cells and nanomolar essential effects on the WT and significant mutant enzymes. Molecule **16** (Figure 2) demonstrated significant antiviral efficacy for the mutants Y181C, K103N, and WT, and these mutants excelled in the cell base experiment with just a slight change in activity between % NHS and % FBS [59]. Dawood et al. produced many new pyrazole and isoxazole-containing heterocycles and identified those with antiviral activity towards herpes simplex type-1. Among these compounds, molecule **17** (Figure 2) showed excellent efficacy and reduced the number of HSV-1 plaques by 69% [60]. Wu et al. introduced the new pyrazole amides with the α-aminophosphonate moiety and praised their antiviral properties. Compound **18** (Figure 2) had some beneficial effects of 50.1% on the tobacco mosaic virus at 0.5 mg/mL [61]. 

Kim et al. discovered novel aryl-substituted pyrazole derivatives as promising non-nucleoside reverse transcriptase inhibitors (NNRTIs) over the virus of human immunodeficiency (HIV) using a cell-based entire replication assay. The enhancement of the antiviral action revealed that molecule **19** (Figure 2), with an EC_50_ of 0.2 nM, had excellent effects on viruses carrying the wild-type HIV-1 as well as viruses carrying the resistance mutations Y181C and K103N in the reverse transcriptase gene [62]. Novel compounds of pyrazole were developed by Ndungu et al., and a SAR method indicated that they were potent measles virus (MeV) inhibitors. Pyrazole **20** (Figure 2) was found to be a potent MeV inhibitor by optimization of its hydrophilicity and in vitro efficacy, with an EC_50_ of 60 nM and hydrophilicity of around 60 μg/mL [63].

Tantawy et al. synthesized a model category of 4-substituted 3-methyl-1,5-diphenyl-1*H*-pyrazoles, and over the kidney cells of Vero African green monkeys, they tested their antiviral efficacy against herpes simplex virus (type-1) in vitro. A control sample was acyclovir. The consequence of the synthetic compounds’ antiviral activity showed that molecule **21** (Figure 2) had superior antiviral activity in comparison to the standard medication (IC_50_ = 0.02) [64]. Zhang et al. developed an extraordinary chain of bis-pyrazole derivatives and tested its effectiveness against the tobacco mosaic virus for antiviral activity (TMV). The findings showed that compound **22** had the same efficacy at 0.1 μg/mL and improved activity more than ningnanmycin at 0.5 μg/mL (Figure 2) [65]. 

**Figure 3:**
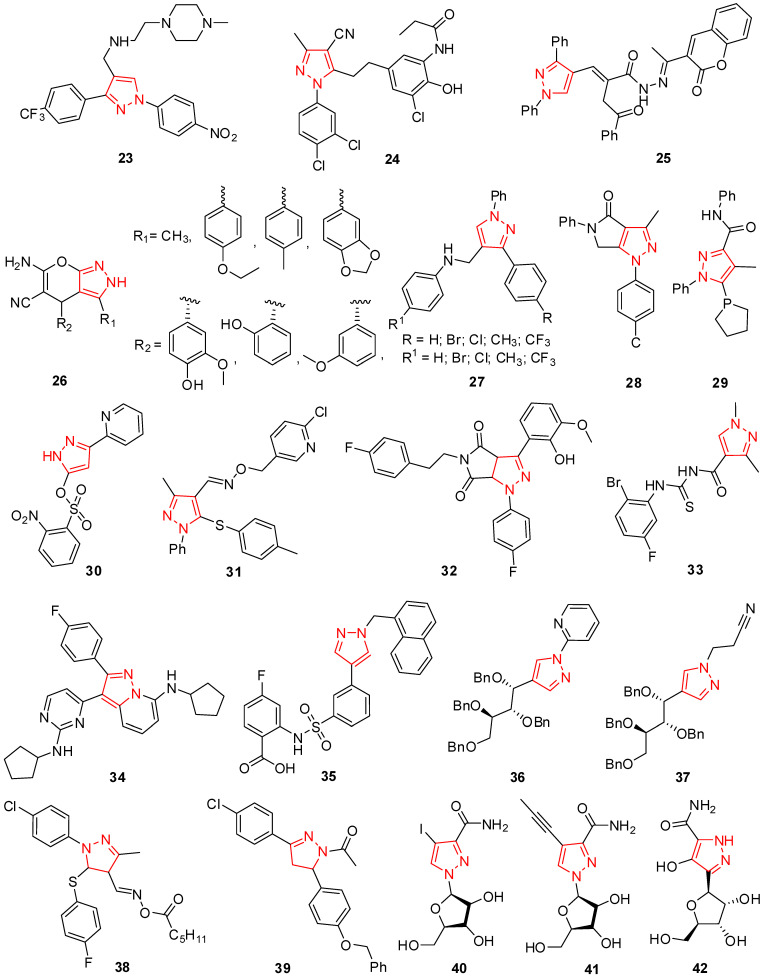
Pyrazole derivatives with antiviral activity.

During phenotypic high-throughput screening employing infectious HCVcc, Hwang et al. identified a class of 1,3,4-trisubstituted pyrazoles as an effective inhibitor of the hepatitis C virus (HCV). Molecule **23** (Figure 3) was the most potent, having an EC_50_ value of 0.11 μM [66].

Mizuhara et al. constructed a novel class of benzylpyrazole analogs to create novel anti-HIV drugs. The 3,4-dichloro derivative **24** (Figure 3), one of the newly created compounds, showed an EC_50_ value of 0.047 μM and significant anti-HIV action [67]. Morsy et al. described the preparation of some prominent new pyrrolone hybrids from which coumarin hybrid **25** presented 90% resistance against NDV [68].

By employing pharmacophore modeling of NNRTIs and 3D-QSAR, Bhadoriya et al. synthesized 24 compounds of diarylaniline derivatives with a pyrazole skeleton that were demonstrated as non-nucleoside reverse transcriptase inhibitors (NNRTIs). 2,4-dihydropyrano[2,3-c]pyrazole (**26**) (Figure 3) was chosen as an NNRTI for anti-HIV-1 chemotherapy from the entire group of compounds [69]. Fioravanti et al. synthesized a class of *N*-((1,3-diphenyl-1*H*-pyrazol-4-yl)methyl)anilines and aggregated them in vitro for antiviral activity and cytotoxicity towards many viruses. Molecule **27** (Figure 3) of the evaluated compounds (EC_50_ = 5–28 μM) inhibited RSV replication at a micromolar dose [70]. 

Many model pyrazole heterocyclic compounds were produced and examined for their anti-BVDV properties and DNA-cleaving catalytic abilities. A fantastic antiviral activity (EC_50_ value 0.12 mmol/L) that was ten times greater when compared to ribavirin (EC_50_ value 1.3 mmol/L) was shown by molecule **28** (Figure 3) [71]. Pyrazolecarboxamide hybrids are a model class of HCV inhibitors, and Manvar et al. developed their inhibition and synthesis process. Molecule **29** (Figure 3) reduced the RNA colony of the contagious Jc1 chimeric 2a clone by 82% at 7 μM and had a selectivity index of **23** and EC_50_ of 6.7 μM against HCV-1b [72]. 

Several pyridine-pyrazole-sulfonate analogs were developed, their effectiveness against HBV was assessed, and the structure–activity relationship (SAR) was discovered in Hep-G2/2.2.15 cells. Molecule **30** (Figure 3) among the sequence had an inhibitory solid action with a high selectivity index (TC_50_/IC_50_) of 35.46 and an IC_50_ value of 9.19 μM [73]. 

The production of pyrazole compounds with antiviral properties and an oxime substrate was reported by Ouyang et al. The findings of the bioassay demonstrated that these compounds had antiviral properties. When compared to the industry reference compound ningnanmycin (EC_50_ = 52.7 g/mL), molecule **31** (Figure 3) was shown to have deactivating capabilities against the tobacco mosaic virus (TMV) (EC_50_ = 58.7 μg/mL) [74]. 

Liu et al. devised a range of pyrrolo pyrazole hybrids and screened each for antiviral efficacy against HIV-1. Compound **32** (Figure 3) is noteworthy for its potent anti-HIV type-1 activity, with an EC_50_ of 3.98 μM and a superb therapeutic index (CC_50_/EC_50_) of more than 105.25. This compound has the potential to be a leading contender for clinical anti-HIV-1 agent optimization. **CC_50_** stands for the half-maximal cytotoxic concentration. It is a measure of a compound’s cytotoxicity, which is its ability to kill cells. The CC_50_ value is the concentration of a compound that kills 50% of a population of cells. A lower CC_50_ value indicates that a compound is more cytotoxic [75]. New pyrazole acyl thiourea analogs were introduced and tested for TMV-inhibitory potential. The therapeutic rates for molecule **33** (Figure 3), one of the novel compounds, were 41.23% [76]. Johns et al. developed pyrazolo[1,5-*a*]pyridine hybrids, which are desired for their antiviral properties against the herpes virus. The most potent antiviral agent was identified as molecule **34** (Figure 3), having an EC_50_ value of 0.26 μM [77].

In the past era, various antiviral agents have been discovered with anti-dengue activity. The *N*-sulfonyl anthranilic acid derivative **35** exhibited good results against dengue virus (Figure 3) [78]. Bari et al. planned the production of diversified C-nucleosides from N and *N*-nucleophiles. These novel molecules were then evaluated against viral strains, including cytomegalovirus (HCMV), herpes simplex virus type-1 (HSV-1-KOS), and human varicella-zoster virus (VZV). Compounds **36** and **37** (Figure 3) persuaded cellular toxicity for the viral strains, but they were deficient compared to the two controls used. Moreover, the lower cytotoxicity of acyclic nucleosides was because of the benzyl-protecting groups, which resulted in low solubility and, hence, low availability of the virus [79].

A new pyrazole **38** (Figure 3) showed a robust antiviral impact against the hepatitis A virus when Hashem et al. produced novel non-nucleoside compounds and evaluated them for anti-hepatitis activity [44]. Starting with α,β-unsaturated ketones, El-Sabbagh and his team synthesized novel *N*-acetylpyrazole derivatives, pyrazolothiazol-4(5*H*)-ones, *N*-thiocarbamoylpyrazole derivatives, and pyrazolothiazole substituents. *N*-acetyl 4,5-dihydropyrazole (**39**) (Figure 3), at subtoxic concentrations with an EC_50_ value of 7 mg/mL, was the most efficient towards the vaccinia virus (Lederle strain) in HEL cell cultures, according to the results of the antiviral screening of these novel molecules against a variety of viruses in different cell cultures [42]. Synthetic ribavirin derivatives with a hydrophobic substituent at the C-4 position were created by Moriyama et al. and showed potential against poliovirus RNA-dependent RNA polymerase. Compounds **40** and **41** (Figure 3) have substantially less potent antipoliovirus action than ribavirin [80]. 

In cell culture, Pyrazofurin **42** (Figure 3) showed antiviral efficacy towards several different DNA and RNA viruses [81]. It was shown to have the same antiviral spectrum as ribavirin and antipox, antirhabdo, antimyxo, antitoga, and retrovirus activity in vitro [82].

## 3. Imidazole Derivatives

Derivatives of imidazole are placed in a unique position in the medicinal chemistry field. The involvement of the imidazole scaffold is a key synthetic strategy in the drug discovery system. The imidazole moiety is a part of several important naturally occurring products, including histamine, purine, nucleic acid, and histidine [83].

**Figure 4:**
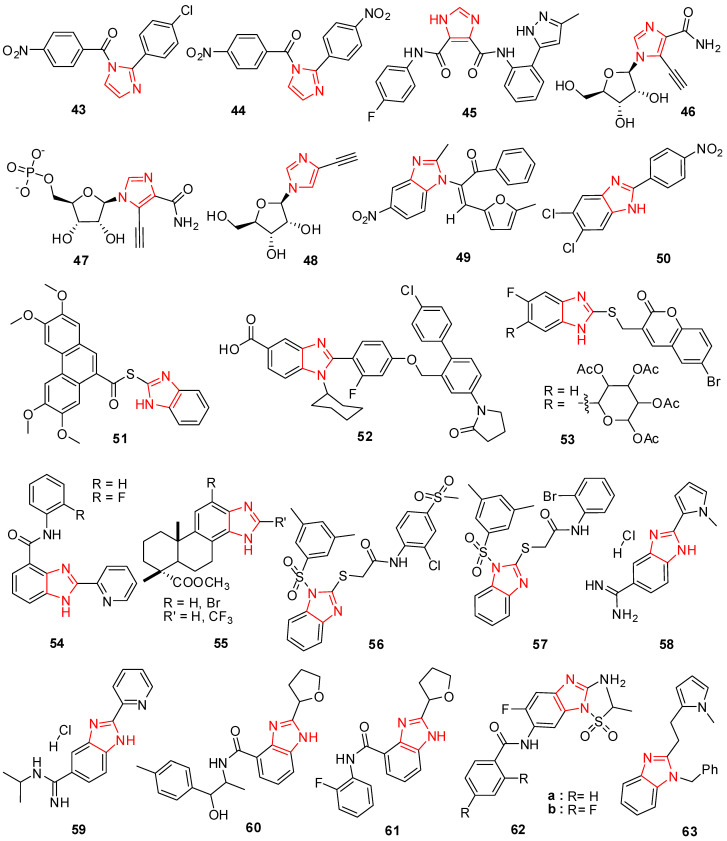
Imidazole derivatives with antiviral activity.

Imidazole products were developed and examined for antiviral activity by Deepika Sharma et al. Molecules **43** and **44** (Figure 4) are superior antiviral agents compared to ribavirin standard medicine, according to the antiviral demonstration of (substituted benzyl)-[2-(substituted benzyl)-imidazol-1-yl]-methanone towards viral strains [84]. HIV-1 protease has been effectively inhibited by imidazole-4,5-dicarboxylic acid (I45DC) and its analogs, including primary or secondary amides [85]. In vitro*,* inhibitory effects on the yellow fever virus (YFV) and the dengue virus (DENV) were evaluated using a new category of imidazole-4,5-dicarboxylic acid (I45DC), which showed micromolar action against both viruses. But with an EC_50_ of 1.85 μM, compound **45** was found to have the highest antiviral efficacy against YFV (Figure 4) [86].

Due to its usage as a medicine, ribavirin has been developed as a lead molecule for many other nucleoside derivatives. De Clercq et al. synthesized a distinct class of 5-alkynyl-1-beta-Dribofuranosylimidazole-4-carboxamides in Figure 4 and identified them as potent antiviral compounds. 5-Ethynyl-1-beta-D-ribofuranosylimidazole-4-carboxamide (EICAR) (**46**) exhibited comprehensive antiviral efficiency against RNA and DNA viruses and presented an antiviral influence about 10–100 times greater than ribavirin (EICAR stands for 5,6-dichloro-1-beta-D-ribofuranosyl-benzimidazole). It is a synthetic antiviral compound that is used as a positive control in antiviral susceptibility testing. EICAR (**46**) is not naturally found in nature and does not cause harm to humans. EICAR (**46**) is used as an antiviral microbe to cure toga-, orthomyxo-, pox-, reo-, paramyxo-, and arenavirus infections [87]. EICAR (**46**) was described to retain an equal range of in vitro antiviral activity but lesser selectivity in contrast to ribavirin [88]. EICAR (**46**) is no longer a therapeutic candidate for managing viral diseases in humans because of its cytotoxicity. EICAR (**46**), nevertheless, has shown effectiveness against animal viruses, such as the epidemic pancreatic necrosis virus, which causes sickness in marine creatures [89], and the canine distemper virus [90]. When converted to 5′-monophosphate (EICARMP) (**47**), it mainly displays as an IMPDH mechanism-based inhibitor [91]. When compared to ribavirin, IM18 (**48**) (1-b-D-ribofuranosyl-4-ethynyl-[1,3]imidazole) reduced the DENV-2 colony in Vero cells by >10 times. Cytotoxic effects were undetectable up to 1000 mM. (IMPDH mechanism-based compounds refer to a class of antiviral compounds that work by inhibiting the enzyme IMP dehydrogenase.) IMPDH is an essential enzyme in the biosynthesis of guanine nucleotides, which are necessary for viral replication [92].

Many benzimidazole hybrids have been produced for their biological activities, such as antiviral activities [93]. Numerous substituted benzimidazoles have either been used in clinical settings or have had their antiviral potency investigated on a range of virus species [94,95]. Shaker et al. developed a model class of 5-nitro-1*H*-benzimidazole analogs by substituting heterocyclic rings at position 1. The antiviral effects and cytotoxicity of the new molecules were investigated. Compound **49** (Figure 4) was the supreme potent compound for its antiviral action towards the adenovirus type-7 strain and rotavirus Wa. Significant antiviral action towards the rotavirus Wa strain was observed with a 70% reduction in contagious rotavirus particles, while adenovirus type-7 displayed a 56.7% reduction [96].

New 2-phenylbenzimidazole hybrids were developed by Michele Tonelli et al., and their cytotoxicity and antiviral action were tested on various DNA and RNA viruses. The compound 5,6-dichloro-2-(4-nitrophenyl) benzimidazole (**50**) outperformed the reference medications 6-azauridine and mycophenolic acid in terms of activity (Figure 4) [97]. 

The phenanthroindolizidine alkaloid antofine (**107**) and its substituents against the tobacco mosaic virus (TMV) showed a remarkable antiviral effect. Yu et al. developed many phenanthrene-holding imidazole compounds and investigated them for anti-TMV action based on the intermolecular interaction between antofine and TMV RNA. The majority of substances showed adequate anti-TMV activity. Several perfect molecules demonstrated superior activity to the market leaders, antofine and ribavirin. Molecule **51** was selected for field studies of its antiviral activity against TMV and showed superior action to the reference plant virus inhibitors [98].

Japan Tobacco (JTK-109) IX (**52**), a benzimidazole with a cyclohexyl ring at position 1, was developed as an inhibitor of the NS5B RNA-dependent RNA polymerase of the hepatitis C virus [99,100].

Hwu et al. presented various new coumarin-ringed benzimidazole hybrids and tested them for hepatitis C virus antiviral activity. Molecules **53** (Figure 4), which were among the products created, were discovered to be effective and had EC_50_ values of 3.4 μM and 4.1 μM, respectively [101].

VERO cells were used by Cheng et al. to test several novel benzimidazoles for their antiviral efficacy against Coxsackie virus B3. In comparison to ribavirin (RVB), which had an IC_50_ value of 411.7 μg/mL and an outstanding selective index greater than 2.42, molecule **54** (Figure 4) from the studied hybrids demonstrated intense selective action with IC_50_ values of 1.43 μg/mL and 0.54 μg/mL [102]. The benzimidazole compounds developed by Fonseca et al. were considered because of their in vitro antiviral efficacy towards several RNA and DNA viruses. From them, molecule **55** (Figure 4) showed commendable action against the varicella-zoster virus and CMV replication and was comparable to ganciclovir and CIDOFO [103].

To create new N1-aryl-2-arylthio acetamido-benzimidazoles, Monforte et al. evaluated compounds based on their type-1 human immunodeficiency virus inhibitory action (HIV-1). Compounds **56** and **57** were determined to be the most potent and least hazardous (Figure 4) [104]. Adenoviruses, Coxsackieviruses, and Echoviruses were tested for antiviral activity by Starcevic et al. using 2-substituted-5-amidino benzimidazoles. From this chain, molecules **58** and **59** (Figure 4) indicated attractive potency against adenoviruses with no cytotoxicity [93]. The anti-Coxsackie virus B3 (CVB3) effectiveness of a few new benzimidazole hybrids was examined in VERO cells by Zhang et al. When compared to ribavirin (IC_50_ = 353.33), molecules **60** and **61** (Figure 4) had impressive inhibitory action with IC_50_ values of 1.06 and 5.30 μg/mL and strong selective indexes of 7.5 and 12.1, respectively [105].

Li et al. synthesized several new benzimidazole structures and tested their ability to suppress the hepatitis B virus. **62a** and **62b** (Figure 4), two of these compounds, have excellent anti-HBV effects, comparable to those of adefovir and lamivudine [95]. The anti-hepatitis B virus (HBV) action of certain new benzimidazole compounds was investigated by Luo et al. Using lamivudine as a reference, compound **63** (Figure 4) showed notable antiviral activity in this study [106].

## 4. Thiazole Derivatives

**Figure 5:**
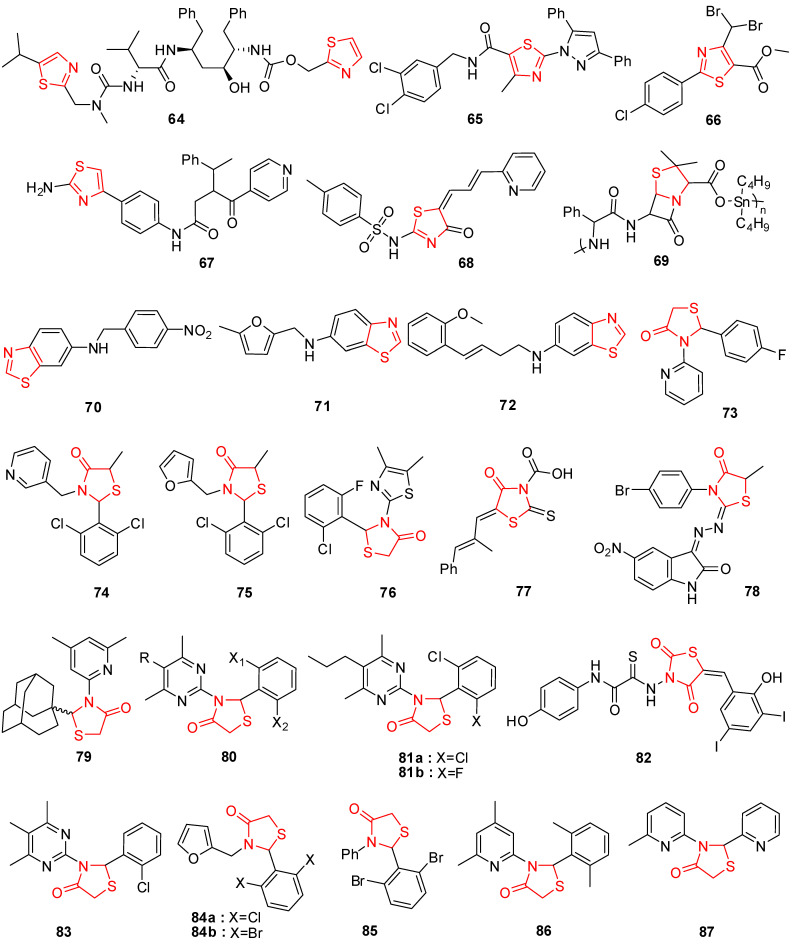
Thiazole and Thiazolidinone derivatives with antiviral activity.

In current periods, the usage of thiazole derivatives is considered in drug synthesis for the medication of HIV infections [107]. Ritonavir (**64**) (bearing thiazole moiety) is an antiviral medication that inhibits the growth of the human immunodeficiency virus (HIV) and causes acquired immunodeficiency syndrome (AIDS) in humans (Figure 5) [108,109]. In cell-based tests, bioassay findings identified several thiazole compounds having antiviral activity. Novel analogs with increased antiviral activity and lower cytotoxicity were developed by altering these lead compounds. In a cellular test system, the most energetic compounds, **65** and **66** (Figure 5), were effective at low micromolar doses [110].

Among the non-nucleoside analogs, thiazole hybrids, particularly BILS 179 BS (**67**) [111] and thiazolones derivative **68** [112], were labeled as prominent antiviral agents against hepatitis C virus (HCV) and HSV, respectively (Figure 5). Within the range of 2 mg/mL, the varicella-zoster virus (VZV), Epstein-Barr virus (EBV), and cytomegalovirus (CMV) were all inhibited by the organotin polymers of ampicillin (**69**) [113].

Host inosine-5′-monophosphate dehydrogenase (IMPDH) is highly active in obstructing the repetition of viruses and is used to produce guanine nucleotides. The effects revealed that N30 (**70**) in vitro inhibited the multiplication of H_3_N_2_, H_1_N_1_, amantadine-resistant strains, and influenza B viruses, including oseltamivir. Systematically, N30 was found to lessen the effectiveness of IMPDH type-II in the neuraminidase inhibition assay and the hem agglutination inhibition assay. Additionally, it was demonstrated that N30 has a potent inhibitory effect on the recurrence of the coronavirus, enterovirus, respiratory syncytial virus, and different strains of Coxsackie B virus [114].

To control their in vitro long-range antiviral efficacy, a family of new unsaturated five-membered benzo-thiazole amine analogs was developed and evaluated. According to the activity data, most targeted compounds showed strong, all-encompassing antiviral effectiveness. Remarkably, molecules **71** with an IC_50_ of 0.71–34.87 μM and **72** with an IC_50_ of 3.21–5.06 μM exhibited strong activity towards both a DNA virus (HBV) and RNA viruses (Cox B3, HCV, and influenza A virus) at low micromolar doses (Figure 5). The SAR demonstrated that aromatic rings with electron-deficient moieties enhanced antiviral efficacy against the viruses of RNA [115].

## 5. Thiazolidinone Derivatives

Thiazolidinones, with a carbonyl group at position 2, 4 or 5, have been subjected to extensive study in recent years [116]. 4-thiazolidinone molecules have great importance due to having anti-HIV [117] and antiviral [118] activities. The efficiency of the 4-thiazolidinone ring structure as an inhibitor of HCV NS5B polymerase has been described by Kaushik-Basu et al. [119].

The HCV NS5B RNA polymerase, an effective target for newly developed anti-HCV medicines, is essential for reproducing the HCV RNA genome. To inhibit HCV NS5B, a brand-new class of 2,3-diaryl-1,3-thiazolidin-4-one hybrids was tried. Out of them, molecules **73**, **74**, and **75** (Figure 5) showed greater efficacy, inhibiting the activity of NS5B RNA polymerase by more than 95% in vitro. The IC_50_ values for the two most effective molecules, **75** and **73**, against HCV NS5B, were 32.2 μM and 31.9 μM, respectively [118]. 

The anti-HIV-1 activity of molecules with thiourea or isothiourea functionality has been very effective. As a result, a class of 2-aryl-3-heteroaryl-1,3-thiazolidin-4-ones was developed and screened for anti-HIV-1 RT activity, and the majority of the compounds from the class exhibited a greater index of selectivity than thiazobenzimidazole (TBZ). Compared to TBZ (EC_50_ = 0.35 μM), the compound 2′-(2-Chloro-6-fluoro-phenyl)-4,5-dimethyl-[2,3′]-bithiazolyl-4′-one (**76**) represented in Figure 5 shows an in vitro EC_50_ of 0.26 μM with low toxicity in MT-4 cells [120]. Hybrids of the 2,3-diaryl-1,3-thiazolidin-4-one category are highly efficient at lowering the cytotoxic effects of HIV-1 on human T-lymphocyte cells [121,122]. Epalrestat (**77**), a hybrid of 2-thioxo-4-thiazolidone, is a strong inhibitor of aldose reductase (Figure 5).

Novel 3-hydrazono-5-nitro-2-indolinone derivatives with substitutions at the thiazolidinone ring were screened against the yellow fever virus and bovine viral diarrhea virus (BVDV). The presence of methyl group at C-5 of the thiazolidinone ring was found to be responsible for measurable levels of antiviral activity. The EC_50_ value of compound **78** was found to be 13 μg/mL against BVDV. Structure–activity relationship (SAR) studies on the thiazolidinone ring revealed that substituents at C-2 and *N*-3 of the ring are responsible for the anti-HIV activity [123]. 

Balzarini et al. studied 2-adamantyl-substituted thiazolidin-4-ones derivatives and found that compound **79** represents an EC_50_ of 0.35 μM against typical non-nucleoside reverse transcriptase. The activity of the compound was due to the presence of an adamantyl moiety at the C-2 position as other compounds lacking the adamantyl moiety were devoid of antiviral activity [124]. 

Thiazolidin-4-ones bearing a lipophilic adamantyl substituent have exhibited good anti-HIV activity and from a series of 2-(2,6-dihalophenyl)-3-(4,6-dimethyl-pyrimidin-2-yl)thiazolidin-4-ones, compound (**80**) exhibited higher anti-HIV activity compared to the other aryl substituted ones [117], where X_1_ = X_2_ = F, X_1_ = X_2_ = Br, X_1_ = X_2_ = Cl, X_1_ = Cl X_2_ = F, R = H, CH_3_.

Chen et al. prepared various 2-(2,6-dihalophenyl)-3-(4,6-dimethyl-5-(un)substituted-pyrimidin-2-yl)-thiazolidin-4-ones and evaluated their HIV-RT inhibitory activity. It was reported that compounds **81a** and **81b** (Figure 5) having an ethyl group at the 5-position on the *N*-3 position of the pyrimidine ring were the most potent ones with IC_50_ values of 0.26 and 0.23 μM, respectively [125].

Ramkumar et al. studied the SAR of rhodanine derivatives and reported that compound **82**, having an electron-donating hydroxyl group at position 4 of ring A and strong electron-withdrawing and hydrophobic substitutions of 3,5-diiodophenol in substructure B, exhibited the best HIV-1 Integrase inhibitory activity with an IC_50_ value of 7 ± 3 μM and 3 ± 2 for 30-processing and strand transfer, respectively [126].

In a series of 2-aryl-3-(4,5,6-trimethylpyrimidin-2-yl) thiazolidin-4-ones, HIV-RT inhibitory activity was greatly affected by substitution at C-5 and enhanced by introducing a chlorine atom on the phenyl at C-2. Compound **83** exhibited remarkable anti-HIV-RT activity (IC_50_ = 2.95 μM) [127].

Rawal et al. evaluated the 2-aryl-3-heteroaryl-2-ylmethyl-1,3-thiazolidin-4-one derivatives for HIV-1 reverse transcriptase (RT) inhibitory activity and found that some of the compounds were effective inhibitors of the HIV-1 reverse transcriptase enzyme at micromolar concentrations with less cytotoxicity. Compounds **84a** and **84b** (Figure 5) showed activity at 0.20 and 0.21 μM concentrations compared to the 0.35 μM concentration of the TBZ-1 lead molecule in MT-4 cells. The activity of this series is dependent on substitution at C-2 and *N*-3 of the 4-thiazolidinone scaffold and a high activity level was observed for compounds that contain a 2,6-dihalophenyl group at C-2 and a pyridine-2-yl or pyrimidine-2-yl ring at *N*-3 [128]. Replacement of the furan ring at *N*-3 with 4-methyl-pyrimidin-2-yl and 4,6-dimethyl-pyrimidin-2-yl **85** was found to enhance reverse transcriptase (RT) inhibitory activity by 1.25–2.5-fold more than nevirapine in MT-4 cells [129]. 

1,3-Thiazolidinone-4-one derivatives were prepared by Ravichandran et al. and their antiviral activity against herpes simplex virus-1 (KOS), herpes simplex virus-2 (G), influenza A H3N2 subtype, and influenza B was evaluated. Compound **86** (Figure 5) was found to be the most active with EC_50_ values of 130.24, 161.38, 249.15, and 263.31 μM, respectively. Compound **87** having a 2-pyridinyl substituent at C-2 and a methyl group at the 6 position of the pyridin-2-yl of *N*-3 of thiazolidinone was found to be the most active with an EC_50_ of 0.0078 μM against wild-type HIV-1 [130].

## 6. Thiadiazole Derivatives

**Figure 6:**
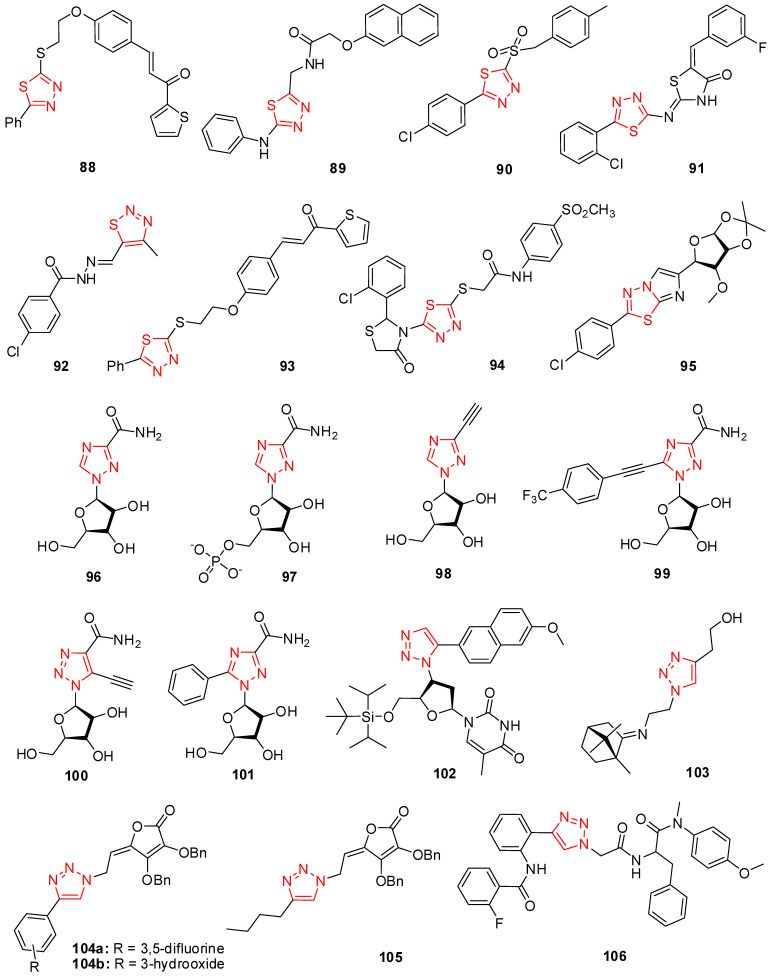
Thiadiazole and Triazole derivatives with antiviral activity.

The nitrogen- and sulfur-containing heterocycles display diverse pharmacological activities which are due to the presence of the thiadiazole moiety and can be evaluated as potential drugs [131,132]. The chalcone-conjugated 1,3,4-thiadiazole compounds were designed and synthesized and their in vitro and in vivo antiviral activities were investigated with microscale thermophoresis (MST) and the half-leaf method. In vitro data indicated that the compounds with phenyl, thiophene, and furan rings showed better antiviral activity than references against the tobacco mosaic virus (TMV), while a substituted phenyl ring which could be EWG or EDG in the chalcone structure was found to be less potent. Compound **88**, represented in Figure 6, demonstrated a high potency against TMV with an EC_50_ value of 30.57 ± 3.11 μM [133].

It is widely known that 1,3,4-thiadiazole compounds have anti-HIV action [134]. According to the MTT assay, Hamad et al. developed thiazole-integrated derivatives. They tested them for in vitro anti-HIV-2 (strain ROD) and anti-HIV-1 (strain IIIB) activity by preventing the virus’s cytopathic effect on human T-lymphocyte (MT-4) cells. The compound 2-(naphthalen-2-yloxy)-*N*-((5-(phenylamino)-1,3,4-thiadiazol-2-yl)methyl)acetamide (**89**) was found to be active among the series with an EC_50_ of 0.96 μg/mL (Figure 6) [135].

Compounds of 5-(4-chlorophenyl)-1,3,4-thiadiazole sulfonamide were tested by Chen et al. for their anti-tobacco mosaic virus efficacy. It was discovered that compounds containing a sulfonamide component were effective tobacco mosaic virus inhibitors with minimal cytotoxicity. A 42% inhibitory compound was 5-(4-chlorophenyl)-*N*-p-tolyl-1,3,4-thiadiazole-2-sulfinamide (**90**), represented in Figure 6 [136].

A series of five-ring *N*-heterocyclic compounds with a halogen-substituted aryl group at the C-5 position of both thiazolidine and thiadiazole derivatives were synthesized as dengue virus (DENV) inhibitors. Among these, the compounds bearing a 3-fluorophenyl substituent at the 4-thiazolidinone core were found to be the most potent compounds. Compounds including 2-fluorophenyl at the C-5 position of the thiadiazole ring and 3-fluorophenyl linked to thiazolidinone exhibited moderate activity with an IC_50_ value of 7.2 ± 0.3 μM against DENV. On the other hand, the SAR data indicated that the replacement of 2-fluoro with 2-chloro as a substituent was linked to 1,3,4-thiadiazole’s (**91**) increased inhibitory activity about 3.5-fold (IC_50_ = 2.1 ± 0.4 μM) [137].

A group of scientists designed and synthesized 4-methyl-1,2,3-thiadiazole-5-carboxaldehyde benzoyl hydrazone derivatives. Some of these derivatives displayed a moderate curative effect against TMV in vivo at 500 μg/mL. The results indicated that compound **92** containing 4-chlorophenyl as a functional group (Figure 6) was found to have the highest curative effect among these derivatives with CE (%) = 49.9 ± 6.3 against TMV [138].

The chalcone-conjugated 1,3,4-thiadiazole compounds were designed and synthesized and their in vitro and in vivo antiviral activities were investigated with microscale thermophoresis (MST) and the half-leaf method. In vitro data indicated that compound **93** (Figure 6) demonstrated a high potency against TMV with an EC_50_ value of 30.57 ± 3.11 μM. It was deduced that the substituted phenyl ring which could be EWG or EDG in the chalcone structure was found to be less potent, whereas phenyl, thiophene, and furan rings enhanced the antiviral activity [133].

The researchers synthesized 2-substituted imidazo [2,1-b] [1,3,4]thiadiazole compounds and their antiviral activity was investigated against the Junin virus (JUNV) in Vero cells. The results stated that the most of obtained compounds showed any inhibitory activity against JUNV but *para*-chlorophenyl-substituted compounds exhibited slight activity and were lower than the reference. Buemi and coworkers synthesized novel non-nucleoside reverse transcriptase inhibitors (NNRTIs). They investigated SARs for 1,3-thiazolidin-4-ones and demonstrated that their NNRT inhibitory effects were dependent on the nature of the conjugated substituents at C-2 and *N*-3 of the thiazolidinone core. The results stated that the compound with an unsubstituted phenyl ring at the C-2 position of thiazolidin-4-one core and that the compound bearing a 3-chlorophenyl-substitution showed the same activity but had lower inhibitory activity than NVP for anti-RT. Compound **94** containing an *ortho*-substituted chloro and methanesulfonyl group (Figure 6) had higher activity [139].

Ribavirin was assessed in Vero cells. The replacement of chlorine with a methoxy group at the 4-position and an additionally substituted methoxy at the 3, 5-position of the phenyl ring led to a decrease in activity. The activity increased by adding a *para*-methoxybenzyloxy- group, and compound **95** containing a chlorine-substituted phenyl ring (Figure 6) had higher activity [140].

## 7. Triazole Derivatives

In 1986, the therapeutic usage of ribavirin (**96**) (RBV), also known as 1-b-Dribofuranosyl-1,2,4-triazole-3-carboxamide (Figure 6), was to treat viral infections, according to a conclusion for hemorrhagic fever and hepatitis C [141]. Ribavirin was presented to deliver a modest inhibitory influence in several cell cultures for multiple mosquito-borne flaviviruses [92,142,143]. When employed as an antiplant viral drug, the inhibitory impact of ribavirin is consistently below 50% at 500 μg/mL [144]. 

For several years, the antiviral agent ribavirin [145,146] has been clinically used to cure infections of respiratory syncytial virus (RSV) in children [147]. Interferon-a and ribavirin have recently become the cornerstone treatments for human HCV illnesses [148]. Ribavirin 5′-monophosphate (RMP) (**97**) in Figure 6 prevents inosine monophosphate dehydrogenase (IMPDH) and was found to be effective for antiviral efficacy toward orthopoxviruses [149], paramyxoviruses, and flaviviruses [150]. Ribavirin has two possible effects after incorporation. It can either inhibit the synthesis or lead to mutations in viral RNA. A virus may experience catastrophic errors due to a concentration of abnormalities in its genomic RNA. This unique mechanism was proposed to explain ribavirin’s antiviral effect against the poliovirus [151]. 

In vitro against flaviviruses (Modoc, Langat, and dengue viruses) [92] and hantaviruses in vivo and in vitro [152], new synthetic 3-ethynyl-1-b-D-ribofuranosyl-1,2,4-triazole (ETAR) (**98**) represented in Figure 6 has recently demonstrated a higher degree of efficacy compared to ribavirin. ETAR decreased DENV-2 replication in Vero cells by ten and showed no harmful effects up to 1000 mM compared to ribavirin [92]. Another binding factor study revealed increased antiviral activity towards HCV by adding a *p*-conjugated system to the 1,2,4-triazole ring-like compound **99** (Figure 6) [153].

Widely used antiviral ribavirin inhibits cellular inosine monophosphate dehydrogenase (IMPDH) and lowers guanosine triphosphate (GTP) levels. Krawczyk et al. presented the medicinal uses of three ribavirin hybrids in this article. Utilizing testing for cytopathic effect prevention in influenza A (H5N1, H1N1, and H3N2), respiratory syncytial viruses, measles, parainfluenza type-3 (PIV-3), and influenza B, some compounds’ antiviral properties were achieved in vitro. These three compounds have modest-to-moderate antiviral activity while inhibiting IMPDH. The 5-ethynyl nucleoside ETCAR (**100**) (Figure 6) showed virus-inhibitory effects against several viruses at concentrations between 1.2 and 20 μM, except for PIV-3, which had weak action at 62 μM. ETCAR’s antiviral efficacy was similar to ribavirin’s, but it had higher cytotoxicity, negatively impacting its selectivity. Bromo or 5-propynyl substitution of the 5-ethynyl group (BrCAR) significantly decreased the antiviral action [154].

Triazoles are a significant class of heterocyclic compounds having different pharmacological activities. Several aryl and alkyl functionalized ribavirin derivatives were synthesized and displayed their antiviral activity against the hepatitis C virus. Among these, compound **101** with an aryl-substituted derivative exhibited the best activity with EC_50_ = 19 ± 0.7 μM against hepatitis C [155].

A group of scientists synthesized a 5′-silylated nucleoside scaffold as an inhibitor against West Nile Virus (WNV) and dengue virus (DENV). Compound **102** with a bulky silyl group shows an inhibition of 74% against WNV and 98% against DENV. It was observed that the presence of cyclohexyl linked to the 5-position of the triazole ring enhanced the activity against both viruses, but the replacement of this group with phenoxymethyl caused a dramatic decrease in activity [156]. 

The researchers synthesized triazole derivatives of 1,7,7-trimethyl-[2.2.1]bicycloheptane by using click chemistry and evaluated their antiviral activity and cytotoxicity against the influenza virus in MDCK cells. Most of these compounds were not toxic for cells but the trimethylsilyl derivative showed the highest toxic effect (CTD_50_ = cytotoxic concentration, the concentration affording 50% death of cells; ED_50_ = effective concentration, the concentration affording 50% inhibition of virus replication). The replacement of the silyl group with an alkyl chain (C2) with hydroxy increased the activity while decreasing the cytotoxicity. Compound **103** (Figure 6) showed a CDT_50_ > 1034.5 μM and ED_50_ of 55 ± 6 μM against H1N1. Further, the antiviral activity enhanced almost 2-fold as a result of the replacement of the hydroxy group with morpholine, while the number of alkyl chains remained constant [157]. 

Two series of 4-substituted-1,2,3-triazole-2,3-dibenzyl-L-ascorbic acid derivatives were synthesized and investigated for their antiviral activity against CMV (cytomegalovirus). Compounds **104a** and **104b** containing electron-withdrawing groups (F and OH) at the 4 positions of triazole rings displayed much better activity against CMV (Davis and AD-160 strains) with EC_50_ = 9.36 μM against the AD-169 strain and EC_50_ = 8.94 μM against the Davis strain. The addition of a long alkyl chain to the triazole C-4 position caused significant changes in the thymidine kinase-deficient (TK-) and thymidine kinase positive (TK+) activities. Compound **105** including a C4 and C9 alkyl chain showed a good potency against TK- with an EC_50_ value of 7.31 μM [158].

A series of 4-phenyl-1*H*-1,2,3-triazole phenylalanine derivatives were synthesized as HIV-1 capsid (CA) inhibitors and most of the derivatives displayed effective antiviral activities in TZM-b1 cells. Compound **106** (Figure 6) with a 2-fluoro group in phenyl caused an increase in activity with an EC_50_ 3.13 ± 0.91 μM [159]. 

## 8. Oxazole and Oxadiazole Derivatives

**Figure 7:**
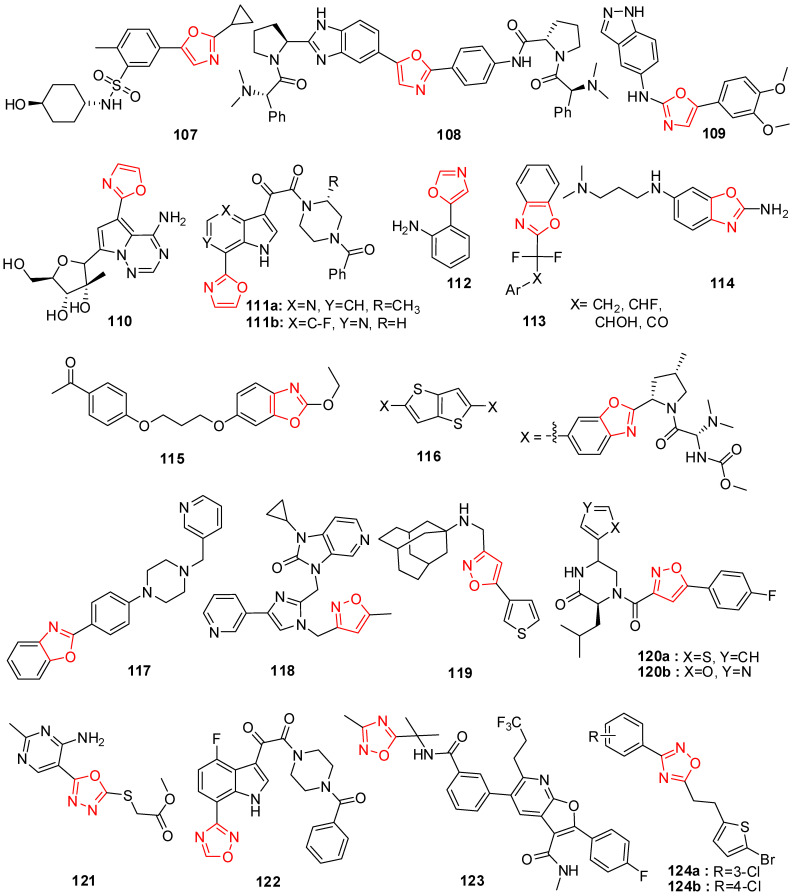
Oxazole and Oxadiazole derivatives with antiviral activity.

Sulfonamide oxazole compounds bearing aminocyclohexanol fragments were designed, prepared, and screened for their PI4KIIIb inhibition which leads to potent inhibition of HCV replication. Compound **107**, represented in Figure 7, exhibited good PI4KIIIb inhibition and importantly was also accompanied by a significant boost in aqueous solubility [160].

Oxazole hybrid compound **108** (Figure 7) bearing a benzimidazole ring exhibited significant HCV genotype-1a (GT-1a) and genotype-1b (GT-1b) inhibitory potency with EC_50_ values of 3.5 and 0.14 nM, respectively. The substitution of the benzimidazole ring by para-substituted analog led to superior inhibition activity in both G-1a and G-1b replicons (EC_50_ = 0.052 and 0.034 mM, respectively) [161].

Bis-substituted oxazole compounds were discovered through cell-based screening of an in-house library and subsequent scaffold modification. Dimethoxy phenyl analog **109** (Figure 7) showed excellent anti-HIV-1 inhibitory activity (EC_50_ = 0.42 mM). The SAR study revealed that anti-HIV-1 activity was quite sensitive to the substituents and their positions on the 5-aryl ring, and the electron-donating methoxy group at the 3-position and electron-withdrawing chloro group at the 4-position were tolerant [162].

Nucleoside inhibitors (NIs) are a valuable class of direct-acting antiviral agents because they often demonstrate broad activity across HCV genotypes and are a high barrier to the emergence of viral resistance. The introduction of an oxazole ring into pyrrolo-triazine yielded compound **110** (Figure 7), resulting in good activity against HCV RNA replication with EC_50_ of 1.8 mM [163]. 

4-Azaindole compounds containing oxazole fragments were synthesized and tested for their anti-HIV-1 activity. Compound **111a** (Figure 7) provided an EC_50_ value of 0.18 mM and was a >10-fold improvement compared to its precursor, and it also exhibited no notable cytotoxicity. Compound **111b** bearing a fluoro atom displayed excellent potency and a good therapeutic index [164].

The study involved synthesizing benzoxazoles and related analogs (**112**, **113**) and investigating their antiviral potential alongside previously developed benzoxazole derivatives (Figure 7). Using a scintillation proximity assay, these compounds were evaluated for their inhibition of reverse transcriptase (RT) activity. The results demonstrated varying IC_50_ values (ranging from 6.3 × 10^5^ µmol/L to 0.34 µmol/L) and highlighted their ability to hinder RT enzyme binding, with a comparison to standard drugs like 3′-azido-2′,3′-dideoxythymidine triphosphate and dideoxythymidine triphosphate. Novel (5-oxazolyl)phenylamine (**112**) derivatives were synthesized and tested for antiviral activity against hepatitis C virus (HCV) and Coxsackie viruses (CVB3 and CVB6) in vitro. Several compounds demonstrated potent antiviral effects against HCV and low cytotoxicity in cells [165]. 

2-Aminobenzoxazoles have been synthesized as ligands for the hepatitis C virus (HCV) internal ribosome entry site (IRES) RNA. In the FRET assay, benzoxazoles **114** (Figure 7) gave excellent antiviral activity with an EC_50_ value of 25 mM. Researchers found that this compound with a flexible substituent at the 6-position might increase the binding affinity to the RNA target. In particular, the presence of the methylene group at the 6-position of the benzoxazole ring was important for the bioactivity [166].

The benzoxazole compound **115** (Figure 7) was synthesized and tested for antirhinovirus activity and resulted in an ID_50_ value of 0.1−0.5 mg/mL. Its protection index was about twice as high as that of Pirodavir on human rhinovirus 14 (HRV14) and up to four times better than that shown by Pirodavir on human rhinovirus 39 (HRV39), and this compound was also more than 60 times less toxic than the reference compound Pirodavir [167].

*C*2-Symmetric bis-benzoxazole analog **116** (Figure 7) with a thienothiophene linker exhibited good antiviral activity against genotype 1b with an EC_50_ value of 0.019 nM. The substitution of one benzoxazole ring by a benzimidazole fragment led to excellent potency against both genotype 1b and 1a replicons (EC_50_ = 0.004 and 0.029 nM, respectively). Structure–activity relationships showed that the excellent potency profiles observed with the thienothiophene scaffold analogs could be maintained when the benzoxazole skeleton was replaced with a range of other heteroaromatic rings [168].

High-throughput screening enzyme-linked immunosorbent assays were employed to evaluate the probable candidates. Benzoxazole analog **117** (Figure 7) displayed good antiprion activity with an EC_50_ value of 0.3 mM, and it was able to penetrate the blood−brain barrier and achieved excellent drug concentrations in the brains of mice after oral dosing [169].

Isoxazoles containing an imidazo[4,5-*c*]pyridin-2(3*H*)-one fragment were prepared and tested against the human respiratory syncytial virus. The results showed that compound **118** gave a two-fold more active activity in comparison with its precursor, while the isomeric isoxazole was four-fold more potent with an IC_50_ value of 41 nM [170].

The effects of isoxazole on the conformation of the S31N TM segment and the dynamics of the proton-selective residue, His37, were investigated by solid-state NMR spectroscopy. Results showed that compound **119** (Figure 7), having a thiazole moiety, exhibited a submicromolar EC_50_ value of 0.353 mM against S31 *N*-containing A/WSN/33 influenza viruses in antiviral plaque reduction assays with a selectivity index greater than 100, indicating that this compound is a promising candidate for in-depth preclinical pharmacology [171].

Piperazinones bearing an isoxazole ring **120a**–**b** (Figure 7) displayed significant GT-1b and 1a potency with EC_90_ values ranging from 0.006 to 0.070 uM, and it was envisioned that the introduction of a heterocyclic moiety at the C-6 position of the piperazinone ring could result in the retention of favorable π−π interactions provided by a C-6 phenyl group [172].

Novel 1,3,4-oxadiazole derivatives containing a pyrimidine moiety were synthesized and evaluated for antiviral activities against tobacco mosaic virus (TMV). The preliminary biological results demonstrated that compound **121** (Figure 7) exhibited an excellent curative effect against TMV with an EC_50_ value of 246.48 mg/mL, which was better than that of Ningnanmycin (EC_50_ = 301.83 mg/mL) [173].

The introduction of an oxadiazole ring into the C-7 position of the indole nucleus (compound **122**) resulted in good antiviral activity against JRFL-envelope-expressing pseudotyped virus with an EC_50_ value of 0.05 nM, and this compound had a 1500-fold better safety index than the C-7 position unsubstituted one [174].

1,2,4-Oxadiazole **123** (Figure 7) bearing a benzofuran ring exhibited good antiviral activity to GT-1a, GT-2a, and GT-1b with EC_50_ values of 9, 4, and 6 nM, respectively. Moreover, this compound exhibited a dose-proportional increase in exposure across the dosing range of 5, 15, and 30 mg kg-1 in rats [175].

1,2,4-Oxadiazoles were prepared and tested for the DENV-2 RdRp assay. Compounds **124a** and **124b** (Figure 7) with chlorine produced superior inhibitory activity with IC_50_ values of 2.2 and 9.1 mM, respectively. Further bioactivity results showed that these compounds exhibited inhibition activities on the replication of dengue virus (DENV1-4) clinical isolates with the EC_50_ in a low micromolar range of 2.0–8.5 mM [176].

## 9. Pyrrole Derivatives

**Figure 8:**
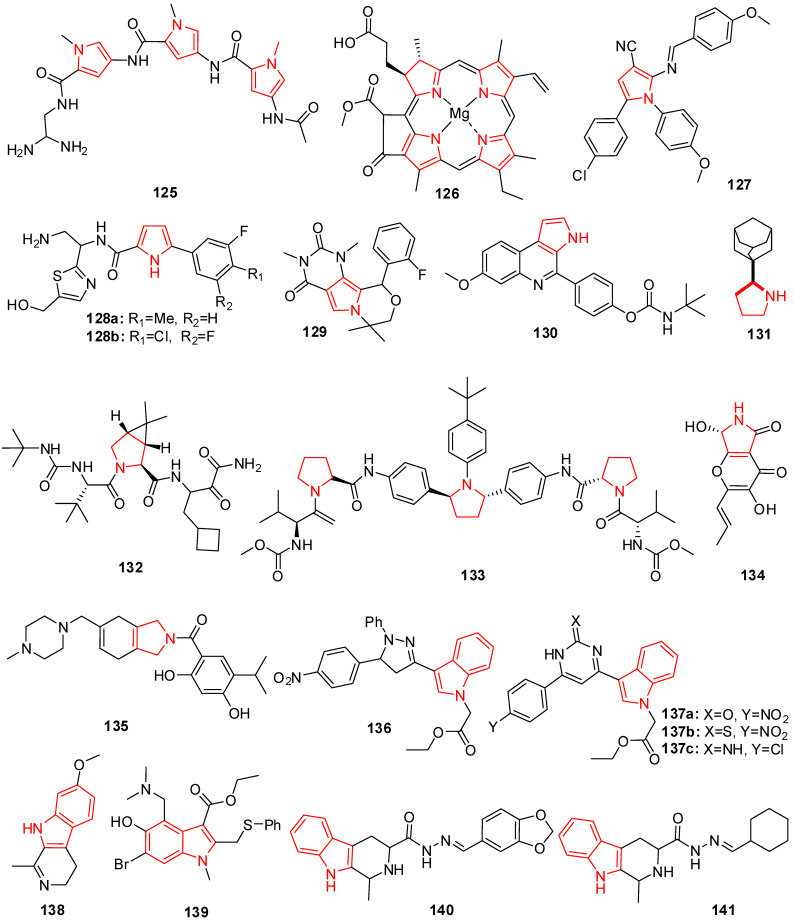
Pyrrole, Pyrrolidine, and Indole derivatives with antiviral activity.

Bisdistamycin (**125**) is a drug-bearing pyrrole moiety that is used as an anti-HIV agent (Figure 8) [177]. A modified authentic immune-absorbance polymerase chain reaction (IA-PCR) test was developed by Lamontagne et al. In this procedure, an anti-HBs antibody mounted on a plate traps viral particles. Real-time PCR is then utilized to evaluate the captured particles’ infection rate directly. This experiment suggested that eight compounds might gradually reduce the amount of released HBV virus particles in the culture medium while having no discernible impact on cell survival. With an IC_50_ of 1.5 μM, the substance chlorophyllide (**126**) (Figure 8) showed the ability to reduce HBV levels by 4–6 times and was chosen for future investigation [178].

The pyrrole analogs were synthesized and compared with acyclovir (ACV), a standard drug used to treat HSV, for anti-HSV activity and cytotoxicity. All new compounds exhibited a high percentage of reduction (94–99%) in the number of virus plaques. Compound **127** had the highest activity (99%) and even had better results than ACV due to their similarity at the substituted N of pyrrole with 4-methoxyphenyl [179].

Curreli et al. have developed drugs to block HIV-1 envelope glycoprotein (gp120) from binding the receptor CD4 of the host cells and prevent the entry of viral RNA. The scaffolds were designed to mimic receptor CD4 and act as HIV-1 entry antagonists. The compounds exhibited high antiviral potency with low cytotoxicity and good selectivity for HIV-1 gp120. (R) **128a** and (S) **128b** (Figure 8) have better selectivity and were used to measure HIV-1 entry antagonist properties with IC_50_ values of 0.45 ± 0.05 in TZM-b1 cells and 0.76 ± 0.03 in MT-2 cells for (R) **128a**. The compounds can also inhibit HIV-1 reverse transcriptase from converting viral RNA into complementary DNA in hosts [180].

Lin et al. introduced a class of anti-influenza agents targeting the viral nucleoprotein (NP), a binding protein that contributes to the transcription and packaging processes. The synthesized pyrimido-pyrrolo-quinoxalinedione analogs were aimed to inhibit the synthesis of NP and interrupt viral replication [181]. The inhibiting effect of compound **129** (PPQ-581) in Figure 8 was compared with that of the nucleozin 3061, a potent antagonist of NP. Both have a similar trend of effectively inhibiting nucleoprotein (NP) synthesis shortly after infection. 

New chemotypes of marinoquinoline derivatives with antiplasmodial activity were discovered and evaluated for their inhibitory activities against *P. falciparum* and a structure−activity relationship study was conducted. The most potent compound **130** with an IC_50_ of 39 nM is a fast-acting inhibitor with dual-stage (blood and liver) activity. The compound showed considerable selectivity (SI > 6410), an additive effect when administered in combination with artesunate, excellent tolerability in mice, and oral efficacy at 50 mg/kg in a mouse model of *P. berghei* malaria with 62% reduction in parasitemia on day 5 postinfection [182].

## 10. Pyrrolidine Derivatives

Saraswati established that oxindole and spiropyrrolidines moiety products experienced against microbial infections were associated with viral and HIV infections [183]. Victrelis (boceprevir) (**132**), a protease inhibitor (Figure 8), was authorized in 2011 by the US Food and Drug Administration for utilization in treating HCV infections together with ribavirin and pegylated interferon [184,185]. Novel series of 2-(1-adamantyl)hexahydroazepines, 2-(1-Adamantyl)pyrrolidines, and 2-(1-adamantyl)piperidines have been developed and validated for their antiviral action toward the viruses of influenza A and B, HIV-1, and HIV-2. Anti-influenza A was evaluated on the size of the ring impact. The anti-influenza virus A action was shown to be significantly reduced by the expansion from a five-membered (pyrrolidine) or six-membered (piperidine) heterocyclic ring to a seven-membered (hexahydroazepine) heterocyclic ring. Rimantadine analog **131** proved to be six times more potent than the Rimantadine drug, while the hexahydroazepine derivatives were inactive [186].

Due to significant advances in antiviral drugs, many pyrrolidines ring-containing analogs are also reported for their potential inhibitory activity toward different viruses. Like telaprevir, ombitasvir (**133**), another antiviral medication, is used as a combination therapy to treat chronic hepatitis C. This molecule inhibits, more specifically, NS5A, a protein essential for viral replication and virion manifestation. This analog also acts as a potent inhibitor of SARS-CoV-2 [187].

Rao et al.’s work based on molecular docking, dynamics simulation, and the screening of small molecules investigated pyranonigrin A (**134**) as a potential inhibitor against the main protease (Mpro) expressed in the SARS-CoV-2 virus [188].

Fakhar et al.’s report based on the structure-based pharmacophore modeling, virtual screening workflow, ADMET, and molecular dynamics simulations revealed that compound **135** is a potential inhibitor of SARS-CoV-2 [189].

## 11. Indole Derivatives

The indole heterocycle displays numerous biological applications, including antiviral activity [190]. Along with antiviral action, indole and its analogs are reported to possess a wide range of other therapeutic characteristics [136,191,192]. Enfuvirtide (T-20; Fuzeon) was the first anti-HIV drug to cure HIV/AIDS and was approved in 2003 by the U.S. FDA. El-Sawy et al. synthesized some new heterocyclic molecules bearing an indole moiety and evaluated their antiviral activity against MDV to minimize the disease course and deaths. Chicken embryo testing showed that the molecules **136**, **137a**, **137b**, and **137c** (Figure 8) exhibit noteworthy anti-MDV activity with substantial therapeutic indices (TIs) of 80–83 and an IC_50_ of 5–6 μg/mL. Cytotoxicity assays showed that the IC_50_ of these compounds was more than 400 and 500 mg/mL [193]. 7-methoxy-1-methyl-4,9-dihydro-3*H*-pyrido [3,4-*b*] indole (Harmaline) (**138**), according to Bag and colleagues, has effective anti-HSV-1 action against both clinical isolates and the HSV-1 wild type (Figure 8) [194]. 

Arbidol (**139**) is an anti-influenza drug (Figure 8), synthesized by the Russian Research Chemical-Pharmaceutical Institute since 1990, and has been used to cure severe respiratory disease and prophylaxis. It displays a comprehensive and practical activity as an antiviral agent against a lot of viruses, including influenza A, B, and C viruses, adenovirus, chikungunya virus [195], hepatitis B [196], and hepatitis C virus [197].

A chain of unique tetrahydro-beta-carboline analogs bearing an acyl hydrazone moiety was planned, produced, and presented for antiviral activity. Most of these molecules demonstrated outstanding activity as both in vivo and in vitro antiviral agents. Some compounds had in vivo inactivation, protective, and curative activities that were significantly greater than those of ribavirin (37.6%, 37.9%, and 39.4%) and the main compound (40.0%, 39.6%, and 42.3%) at 500 μg/mL. Molecule **140** (Figure 8) had in vitro and in vivo values of 36.9%, 33.6 percent, 30.2%, and 35.8% at 100 μg/mL, which were highly similar to ribavirin’s in vitro activity of 40.0% at 500 μg/mL. According to the results, molecule **141** (Figure 8) had improved activity compared to control plant virus inhibitors and was approved for field tests of antiviral effectiveness against TMV (tobacco mosaic virus) [198].

## 12. Isatin Derivatives

**Figure 9:**
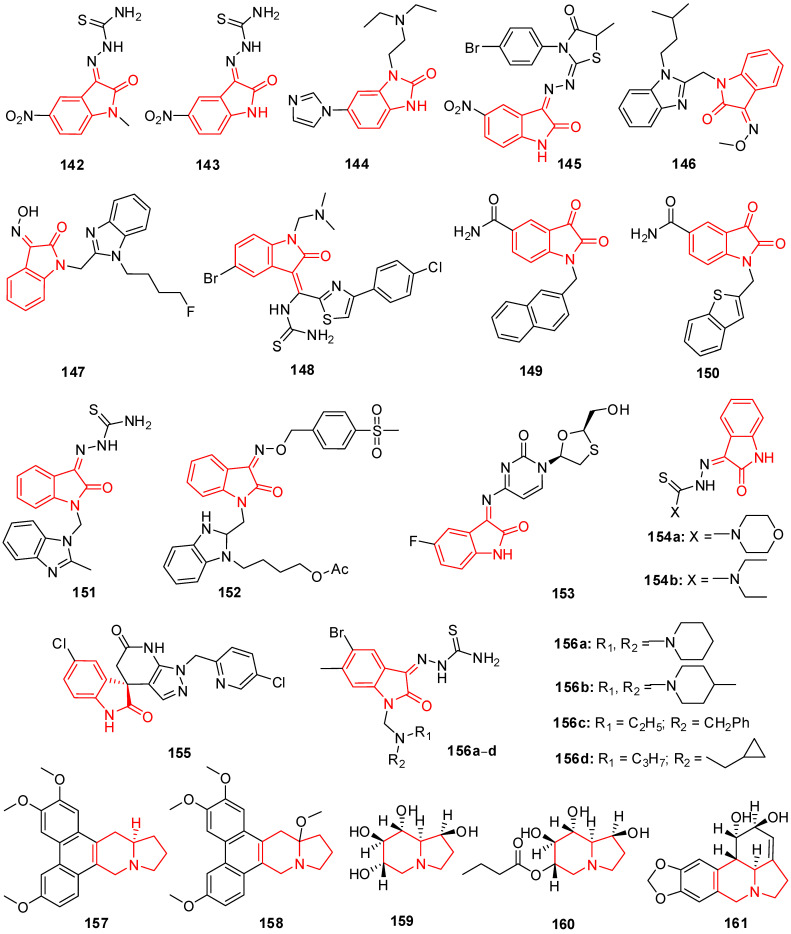
Isatin and Indolizidine derivatives with antiviral activity.

Certain thiourea and semicarbazone analogs have been identified as having antiviral properties, and these properties are linked to the existence of entire NHC(=S)NH and NHC(=O)NH groupings [199]. Numerous scholars have reported the antiviral properties of isatin- β-thiosemicarbazone (1*H*-indole-2,3-dione-3-thiosemicarbazone) and its *N*-Mannich bases [200,201]. Methisazone (*N*-methylisatin-β-thiosemicarbazone (**142**) and isatin-β-thiosemicarbazone (**143**)) was the first clinically approved antiviral agent, actively used against poxviruses [202]. It has been determined that the cyclic urea analog-like compound **144** (Figure 9) inhibits the virus replication that causes bovine viral diarrhea [203].

Terzioglu et al. developed a novel result of 5-nitro-3-[(5-nonsubstituted/methyl-4-thiazolidinone-2-ylidene)hydrazono]-1*H*-2-indolinone derivatives. The main antiviral activities of the new hydrazonoindolinone analogs, 5-nitro-1*H*-indole-2,3-dione-3-thiosemicarbazones, and 1-morpholino/piperidinomethyl-5-nitroindole-2,3-dione-3-5-nitro-1*H*-indole-2,3-dione-3-thiosemicarbazones were screened towards specific toxic pathogens. Some of the examined substances inhibited the growth of the BVDV in MDBK CODA cells. When compared to ribavirin (EC_50_ of 40 μg/mL), the antiviral activity of molecule **145** (Figure 9) against BVDV was good (EC_50_ of 13 μg/mL) [123].

(Z)-1-((1-isopentyl-1*H*-benzo[d]imidazol-2-yl)methyl)-3-(methoxyimino)indolin-2-one (**146**) in Figure 9 showcases applications that are efficient against various viruses. Isatinoxime ethers have been used for in vitro research because of their inhibitory action and cytotoxicity against the respiratory syncytial virus (RSV), which infects and kills newborns [204]. Respiratory syncytial virus illnesses stimulate children’s asthma. Compound (E)-1-((1-(4-fluorobutyl)-1*H*-benzo[d]imidazol-2-yl)methyl)-3-(hydroxyimino)indolin-2-one (**147**) has proven to be active against the infection of respiratory syncytial virus in in vivo and in vitro studies (Figure 9) [204]. 

Isatin Schiff base ligands were developed by Pandeya et al. by reacting 5-halo isatin with *N*-[4-(4′-chlorophenyl)thiazol-2-yl]. These compounds were transformed into thiosemicarbazide and their *N*-Mannich bases by reactions with formaldehyde and three secondary amines. All substances show antimicrobial activity, which was evaluated for anti-HIV activity towards HIV-1 (IIIB) replication in MT-4 cells. They include the substance 1-[*N*,*N*-dimethylaminomethyl] 5-Bromo isatin-3-(1′-(4″-(p-chlorophenyl) thiazol-2″-yl) thio semicarbazone) (**148**) represented in Figure 9, which was found to have the most effective behavior for treating HIV [205].

A series of *N*-substituted isatin derivatives have been designed and synthesized to act as potent inhibitors of the SARS-CoV-2 main protease. The enzyme inhibition activity of compounds was evaluated using synthetic peptide-pNA as the substrate and tideglusib as a positive control. Compound **149** (Figure 9) was the most promising candidate (IC_50_ = 0.045) [206].

Isatin derivatives were synthesized and examined for their potential inhibitory effects on purified HRV-14 3CP. Compounds bearing carboxamide groups at C-5 are far more potent than those carrying other groups. In addition, compounds substituted at *N*-1 with larger alkyl groups are 2–25-fold more potent than those bearing smaller alkyl groups. Compound **150** (Figure 9), bearing a benzothiophene group, was identified as the most potent analog with *Ki* bearing a benzothiophene [207].

Mishra and his coworkers synthesized a hybrid molecule (MBZM-*N*-IBT) of 2-methyl benzimidazole and isatin-β-thiosemicarbazone and evaluated it for its anti-chikungunya virus activity. Compound **151** (Figure 9) displayed enhanced antiviral activity as it significantly inhibited chikungunya virus replication. In mammalian systems, the following events were identified: (i) a reduction in viral particle formation by >75%, (ii) a decrease in viral RNA synthesis by >65%, and (iii) a reduction in viral protein levels by >97%. Molecular modeling studies for this compound revealed strong binding affinities to the structural and nonstructural proteins of the chikungunya virus and related viruses. In silico pharmacokinetic prediction indicated favorable drug-like properties [208].

The benzimidazole-isatin oximes were synthesized and tested for anti-RSV activity which demonstrated excellent antiviral activity (EC_50_ = 5–100 nM). Some compounds were identified as the most potent derivatives relative to BMS-433771. Compound **152** emerged as the most efficacious with an EC_50_ value of 5 nM. The SARs indicate a good correlation with that reported for the benzimidazole-2-one series [209].

Different isatin-lamivudine hybrids were synthesized by Sriram et al. and evaluated against HIV-1 in a CEM cell line. The results showed compound **153** to be equipotent to lamivudine with an EC_50_ of 0.0742 µM, CC_50_ of >200 µM, and SI of >2100. The SARs demonstrated that the introduction of -F at the C-5 position of isatin boosted the activity (EC_50_ = 0.0742–1.16 µM) and resulted in less cytotoxic compounds (CC_50_ = 123–>200 µM), whereas -Cl was detrimental to activity (EC_50_ = 4.73–>12.5 µM) and increased toxicity (CC_50_ = EC_50_ = 4.73–12.5 µM), in the CEM cell lines [210]. 

Kang et al. designed and synthesized a series of isatin-β-thiosemicarbazones which were evaluated for antiviral activity against HSV-1 and HSV-2, and acyclovir was used as a positive control by utilizing the plaque reduction assay. Compound **154a**, which bears a morpholine group, was identified as the most potent derivative against HSV-1 (IC_50_ = 1.3 µM) with a cytotoxicity of >25 µM. Furthermore, it was able to inhibit HSV-2 at a concentration of 2.74 µM (IC_50_ of acyclovir was 1.27 µM). Notably, compound **154b** demonstrated potent and selective inhibition against HSV-2 (IC_50_ = 1.54 µM) [211]. 

Adopting high-throughput phenotypic screening, Zou et al. synthesized a series of spiropyrazolopyridones as a new class of anti-DENV agents. Various compounds showed promising antiviral activity against DENV-2, with compound **155** being the best with an EC_50_ value of 0.011 µM [212]. 

A series of isatin-β-thiosemicarbazones was synthesized and examined for antiviral activity and cytotoxicity in vaccinia virus- and cowpox virus-infected human cells using methisazone and cidofovir as positive controls. Compounds **156a**–**d** (Figure 9) did inhibit cowpox virus plaque formation (EC_50_ = 6 ± 2.9–6.2 ± 1.6 μM with SI > 8) with greater potency than methisazon and cidofovir. Concerning their activity against vaccinia virus plaque formation, these compounds exhibited enhanced efficacy (EC_50_ = 0.6 ± 6.8 μM with SI > 35) and were more potent than the standard drug cidofovir. Moreover, compounds **156a, c,** and **d** showed better activity in vaccinia virus plaque formation inhibition than methisazone (EC_50_ = 3.3 ± 3.2 μM). The SARs identified 5-bromoisatin as a key pharmacophore for better antiviral efficacy in the designed library. Compound **156d** carrying acyclic secondary amine emerged as the most potent analog against both tested strains. As potent and much more selective antipoxvirus isatin-β-thiosemicarbazone derivatives were identified, further drug discovery processes are warranted [213].

## 13. Indolizidine Derivatives

Whether an extract from Cyanchum komarovii’s aerial parts had exceptional antiviral activity towards TMV was investigated. The main chemical components of *Cyanchum komarovii* that were active were the phenanthroindolizidine alkaloids, with a high concentration of antofine (**157**) in Figure 9 [214]. Antofine was shown to have much more inhibitory action than the marketed antiviral drugs DADHT and DHT [215,216]. The synthesis of many antofine analogs through investigations on the structure–activity connection revealed that the presence of the phenanthrene ring and the tertiary amine are essential for strong antiviral activity [217,218,219]. Antiviral mechanism research revealed that antofine and the origin of TMV RNA (piRNA) act in a potentially beneficial way to suppress the spread of the virus by preventing the commencement of virus assembly [220].

The efficacy of many phenanthroindolizidine 1alkaloid derivatives to restrict tobacco mosaic virus (TMV) RNA in vitro and in vivo was investigated. The majority of compounds in this class were shown to be good-to-exceptional antiviral agents towards TMV compared to the control utilized. The bioassay findings showed that when combined with Ningnanmycin, molecule **158** represented in Figure 9 displayed superior inhibition (77.6% at 500 μg/mL and 55.6% at 100 μg/mL) and exceptional in vivo efficacy. Some molecules exhibited more potent activity at both levels for the curative effect and inactivation effect than commercial Ningnanmycin. Structural activity relationships explained that the 13a position with hydrogen donor substituents was labeled to be promising for exhibiting excellent antiviral activity [221].

An indolizidine alkaloid, castanospermine (**159**), was reported to have strong repressive action against DENV. When tested in both Huh-7 and BHK-21 cells, it inhibited DENV-2 with IC_50_ values of 85.7 μM and 1 μM, respectively. Castanospermine was also able to inhibit other DENV serotypes. It was noted that this alkaloid affected the DENV virus-like particle (VLP) and reduced the DENV particles by more than 95%. Castanospermine **159** (Figure 9) also showed promising antiviral activity against DENV infection in a mouse model [222].

Another in vivo study involving 6-*O*-butanoyl castanospermine (**160**) alkaloid used a zosteriform model in mice. Oral treatment with this alkaloid delayed the development of lesions caused by the SC-16 strain of HSV-I. Additionally, the amount of the virus isolated from the brain of mice was also decreased. In comparison to untreated controls, the viral load inside the brain tissue of mice who received treatment 2 days before infection was reduced by a factor of 100. The IC_50_ of **120** against HSV-I was 15 ± 4.8 μM when given before infection and 37 ± 5.5 μM when given after [223].

The lycorine alkaloid (**161**, Figure 9) showed a potent inhibition of the Zika virus with an EC_50_ value of 0.41 μM and an SI of 35.4 against this virus. The lycorine alkaloid also showed significant inhibitory activities in Vero cells against PV by reducing the CPE caused by this virus. The viral CPE of poliovirus on VERO cells was strongly suppressed at doses of 2.5 μg/mL [224].

## 14. Imidazo-Pyrimidine Derivatives

GBBR is used for the one-pot synthesis of therapeutically relevant fused imidazoles bridgehead nitrogen heterocyclic compounds. The GBBR products can be used for the synthesis of a variety of more complex scaffolds and have diverse applications in combinatorial and medicinal chemistry and its products are of great use in drug discovery [225].

**Figure 10:**
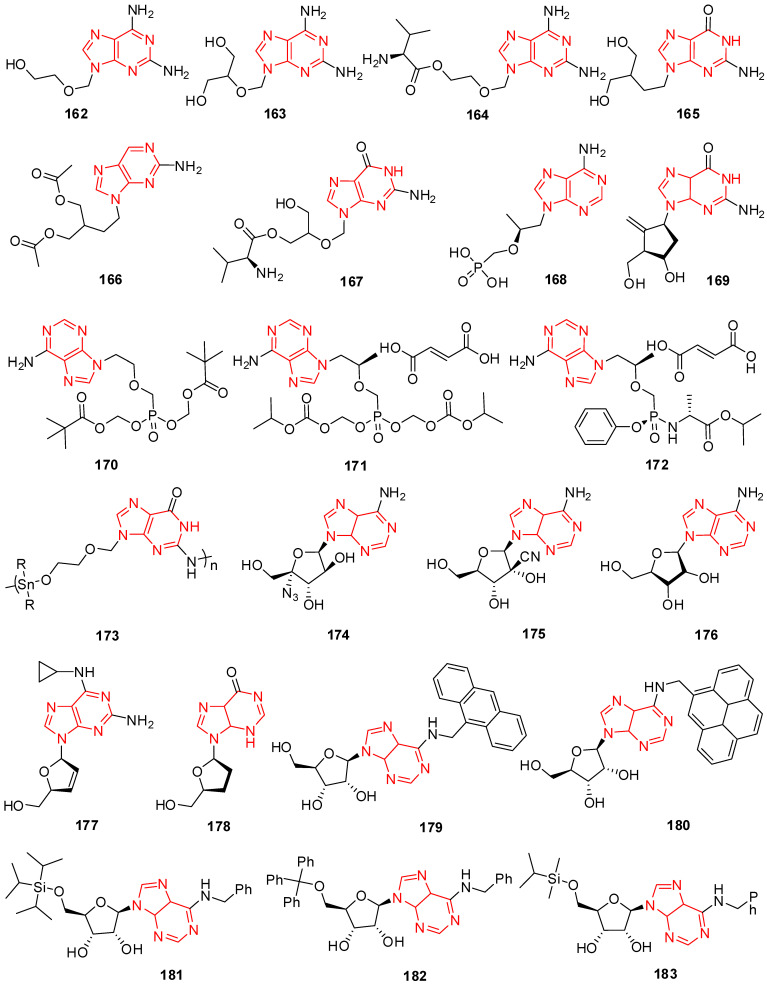
Imidazopyrimidine derivatives with antiviral activity.

Acyclovir (**162**) is widely used to prevent several herpes infections, especially HSV-1 and 2 (Figure 10). Additionally, it is used to prevent the cytomegalovirus (CMV), varicella-zoster virus (VZV), and Epstein-Barr virus (EBV). Acyclovir has a particular inhibitory action. This makes it a first-line antiviral medication [226]. Acyclovir is a potent nucleoside that plays an essential biological role in all living matter and functions [227].

The other acyclic nucleosides (Figure 10) such as ganciclovir (GCV) (**163**), valaciclovir (VACV) (**164**), penciclovir (PCV) (**165**), famciclovir (FCV) (**166**), acyclovir (ACV) (**109**), and valganciclovir (**167**) (VGCV) have been used against herpes viruses [228,229]. Various nucleoside derivatives were tested against the dengue virus, and the results showed that the inhibition effects of valganciclovir (**153**) (VGCV) may be studied further as an anti-dengue agent and leader in the production of new derivatives [230]. Tenofovir (**168**) was the best antiviral agent against HIV/AIDS [231,232]. 

The 2′,3′-dideoxynucleosides (ddNs) have been demonstrated as the most potent therapeutics for HIV because the termination of DNA can occur in the absence of the 3′-hydroxyl group arrangement, such as Zalcitabine (ddC), Stavudine (d4T), and Zidovudine (AZT). Entecavir (ETV) (**169**) [233,234] and Adefovir dipivoxil (ADV) (**170**) have been permitted as inhibitors of reverse transcriptase for the treatment of HBV. L-nucleosides such as Lamivudine (3TC) and acyclic nucleoside prodrugs such as Tenofovir disoproxil fumarate (TDF) (**171**) and Tenofovir alafenamide (TAF) (**172**) [235] have been used as inhibitors of reverse transcriptase for the cure of HBV and HIV (Figure 10). 

The acyclovir organotin polymers (**173**) (Figure 10) revealed the suppression of the Epstein-Barr virus (EBV), cytomegalovirus (CMV), and varicella-zoster virus (VZV), with a range of 2 mg/mL [113]. 

In vitro*,* antiviral activity towards TBEV was reported in the 4′-azido modified nucleoside analog 4′-azido-aracytidine (RO-9187) (**174**) [236]. 2′-C-Ethynyl modified nucleosides have primarily been used as DENV inhibitors [237,238,239,240,241]. The principal compound in these sequences, 2′-C-ethynyladenosine (**175**) in Figure 10, reduced DENV-2 replication while conducting cell-based research using CC_50_ values of 40 mM and 1.41 mM [237]. 

Vidarabine (**176**) [242] has been utilized as a DNA polymerase inhibitor for the cure of herpes viruses. The 2′,3′-dideoxynucleosides (ddNs) have been demonstrated as the most potent therapeutics for HIV because the termination of DNA can occur in the absence of the 3′-hydroxyl group arrangement, such as Abacavir (ABV) (**177**) [243] and Didanosine (ddI) (**178**) [244].

When evaluated against TBEV, N6-aryl or alkyl-modified nucleosides (**179**–**183**) (Figure 10), which were previously determined to be antagonistic of enterovirus A, Lassa fever virus, and Marburg virus, give potent bioactivity [245,246,247]. N6-substituted derivatives of 5′,2′ and 5′,3′-O-tert-butyldiphenylsilyl-modified adenosine also showed mild anti-DENV and anti-YFV activities [248].

## 15. Pyrimidine Derivatives

**Figure 11:**
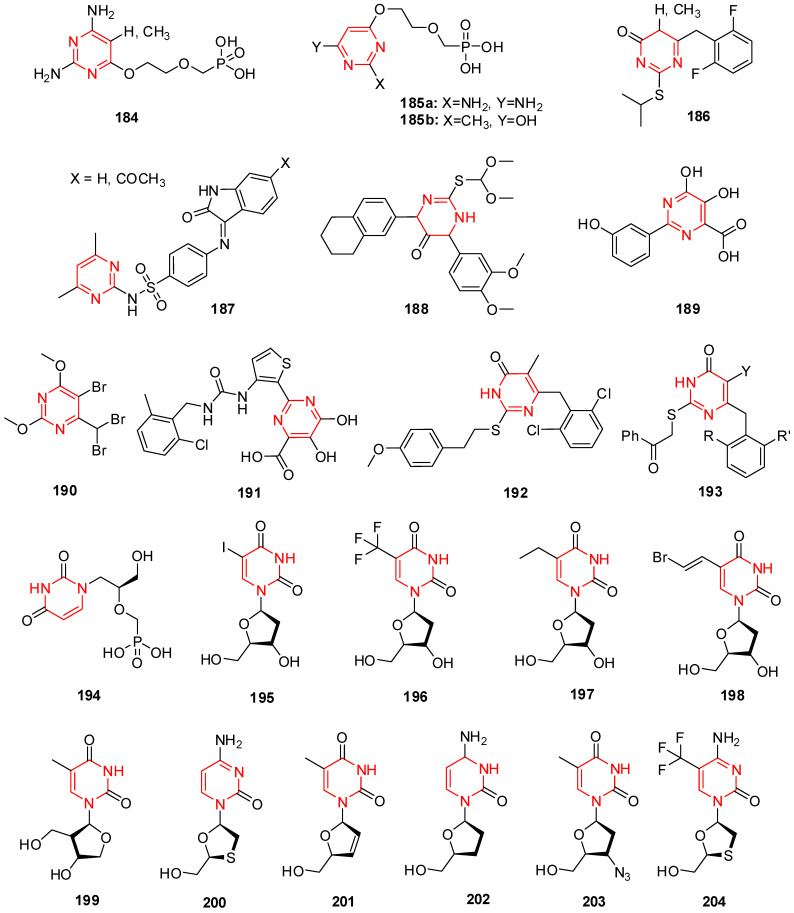
Pyrimidine derivatives with antiviral activity.

The literature survey described that the compounds containing a pyrimidine moiety show pharmacological activities like antiviral activity [249]. Pyrimidines demonstrate biological activity that inhibits several distinct viruses in vitro, including the herpes virus [250], poliovirus [251], and HIV [252,253,254]. Hockov’a et al. produced the 2,4-diamino-5-cyano-6-pyrimidine hybrids (**184**, Figure 11) and tested them for their antiviral properties in vitro. They demonstrated significant activity towards the *cytomegalovirus*, herpes simplex type-1 or 2, and vaccinia viruses, with inhibitory values ranging from 0.0027 μmol/mL to 0.011 μmol/mL [255].

The antiviral potential of 6-[2-(Phosphonomethoxy) alkoxy]pyrimidine substituents was reported by Hol’y et al. These analogs show that antiviral action varied across different molecules, and molecules with amino groups at the C-2 positions of the pyrimidine ring like **185a** and **185b** proved to be the most effective ones [256]. To effectively treat AIDS, Sriram et al. developed the aminopyrimidinimino isatin derivatives, a new nonnucleoside reverse transcriptase inhibitor with a range of chemotherapeutic characteristics. 

In 1999, novel enzyme-based and cell-based assays were developed and used to investigate the 5-alkyl-2-(thio)-2,6-(-diphenylmethyl)-3,4-dihydropyrimidine-4(3*H*)-one analog as an anti-HIV drug. The results showed that molecule **186** (Figure 11) with the 6-(2,6-difluorophenylmethyl) substituent was the most effective, which was much higher than that of nevirapine, with an EC_50_ value of 40–90 μM [257]. Selvam and coworkers developed the 4-[(1,2-dihydro-2-oxo-3*H*-indole-3-ylidene)amino]-*N*-(4,6-dimethyl-2-pyrimidinyl)-benzene sulfonamide in consideration of the impact of sulfonamides and tested it for anti-HIV activity, and they revealed that molecule **187** (Figure 11) was more effective in membrane type-4 [MT-4] against HIV-1 and HIV-2 replication [258]. 

The 2-(2,4-dioxopentan-3-ylthio)-1,6-dihydro-4-(1,2,3,4-tetrahydronapthalen-6-yl)-6-(3,4-dimethoxyphenyl)pyrimidin-5(4*H*)-one analog was produced by Mohamed et al., and its in vitro antiviral activity allowed it to be recognized. Molecule **188** (Figure 11) was shown to be an incredibly advantageous compound when compared to acyclovir and was acknowledged as a fantastic antiviral drug with 90% inhibition [259]. 

Summa et al. developed a model sequence of dihydroxypyrimidine carboxylic acid analogs, and the related *N*-methylpyrimidinone was subsequently examined using the HCV replicon assay. The potent molecule **189** (Figure 11), which has the functionality of phenol at the meta position, has an IC_50_ value of 5.8 μM [260]. 

Prekupec et al. generated new acyclic, substituted pyrimidine analogs with fluorine at position 6. They tested them for their in vitro antiviral action towards the following viruses: varicella-zoster, parainfluenza virus-3, Punta Toro virus, Sindbis, retrovirus-1, and *cytomegalovirus* (CMV AD-169 and Davis strain) in human embryonic lung (HEL) cells. The analog of 5-bromopyrimidines **190** showed outstanding efficacy (Figure 11) [261].

The 2-(2-thienyl)-5,6-dihydroxy-4-carboxypyrimidine analogs were tested as inhibitors of NS5B for the virus of hepatitis C, which were developed by Koch et al. Molecule **191** (Figure 11) showed an intriguing cell-based action that was 60 times better than an experiment using an enzyme and had no cytotoxicity up to 100 μM [262]. Dihydroxy-alkoxybenzyl-oxypyrimidines (DABOs) were prepared using a microwave, and Manetti et al. identified them based on their antiviral efficacy against highly purified recombinant wild-type and mutant HIV-1 strains as well as the HIV-1 reverse transcriptase (RT) enzyme (wild-type and mutants). Molecule **192** (Figure 11) demonstrated an anti-HIV profile equivalent to efavirenz and nevirapine, according to data from an anti-HIV reverse transcriptase test and anti-HIV efficiency in lymphoid cells [263]. 

Nawrozkij et al. developed many dihydro-alkylthio-benzyloxopyrimidines and examined the compounds’ in vitro antiviral efficacy. Due to their capacity to suppress the 50% HIV-1 strain, compound **193** (Figure 11) with ethyl, isopropyl, and 2,6, dichloro and 2,6, difluoro substitutions at C-5 and C-6 were found to be the most potent analogs [264]. Cidofovir (**194**) is used to cure cytomegalovirus (CMV) [265].

Currently, there are approximately 25 pharmaceutical nucleosides that have been authorized for use in treating highly significant viral diseases. Many nucleoside analogs have been reported to suppress flaviviruses that are transmitted by arthropods. Anti-HCV nucleoside analogs provide intriguing weapons to be utilized towards additional Flaviviridae family viruses that strongly correlate these viruses with the hepatitis C virus (HCV), for which multiple potent medicines are currently being developed [22].

Although interferon-a and seven other nucleoside/tide compounds (NAS) have been authorized for use in the therapy of chronic hepatitis B, these therapies are indeed ineffective and not “curative” [266]. This method has already been effective in finding several possible treatments for the hepatitis C virus (HCV) [267]. Human immunodeficiency virus type-1 (HIV-1), varicella-zoster virus (VZV), human hepatitis B (HBV) and C (HCV) virus, human cytomegalovirus (HCMV), herpes simplex virus (HSV), and other disorders have all been treated with nucleoside derivatives [268]. Currently, more than 20 nucleotide and nucleoside derivatives expressed in Figure 11 have received FDA approval for use as antiviral medications for a variety of illnesses, including diverse viral hepatitis, HIV infection, herpes virus infections, and acquired immune deficiency syndrome (HIV/AIDS) [268].

The first antiviral medication used was Idoxuridine (5-iodo-2-deoxyuridine) (IDU) (**195**). It has been used extensively to treat viral illnesses. In 1961, Herrmann reported its antiviral activity against HSV [269]. Idoxuridine IDU is used to treat genital herpes eye damage topically. It was discovered that trifluridine (5-trifluoromethyl-2-deoxyuridine) (**196**) was effective against viruses resistant to IDU treatment [270]. Trifluridine (TFT) [271], 2′-deoxynucleosides as Idoxuridine (IDU) (**121**) [269], Edoxudine (EDU) (**197**) [242], and Brivudine (**198**) [272] (BVDU) have been utilized as DNA polymerase inhibitors for the cure of herpes viruses. Telbivudine (LdT) (**199**) has been permitted as an inhibitor of reverse transcriptase for the treatment of HBV [273]. 

Lamivudine (**200**) is an active antiviral agent against hepatitis B [233,234]. When used with zidovudine, lamivudine is also an efficient anti-AIDS agent [265]. Stavudine (**201**), another pyrimidine nucleoside, possesses high inhibition against the dengue virus. Its inhibitory effects against dengue declare it the lead compound for new analogs against dengue infections [230]. Stavudine, when combined with zidovudine, is thought to have notable anti-HIV efficacy [274].

Along with zalcitabine (**202**), zidovudine (**203**) is an effective drug [275]. The dideoxyribose molecule of the thymidine derivative zidovudine, which has an azido group at the third position, is effective in the retroviruses that cause AIDS and T-cell leukemia (Figure 11). As a significant inhibitor of HIV’s in vivo replication and cytopathic effects, it has been recognized to be utilized in the AIDS treatment and severe AIDS-related complex (ARC) [276]. L-nucleosides such as Emtricitabine (**204**) (FTC) have been used as inhibitors of reverse transcriptase for the cure of HBV and HIV [235].

**Figure 12:**
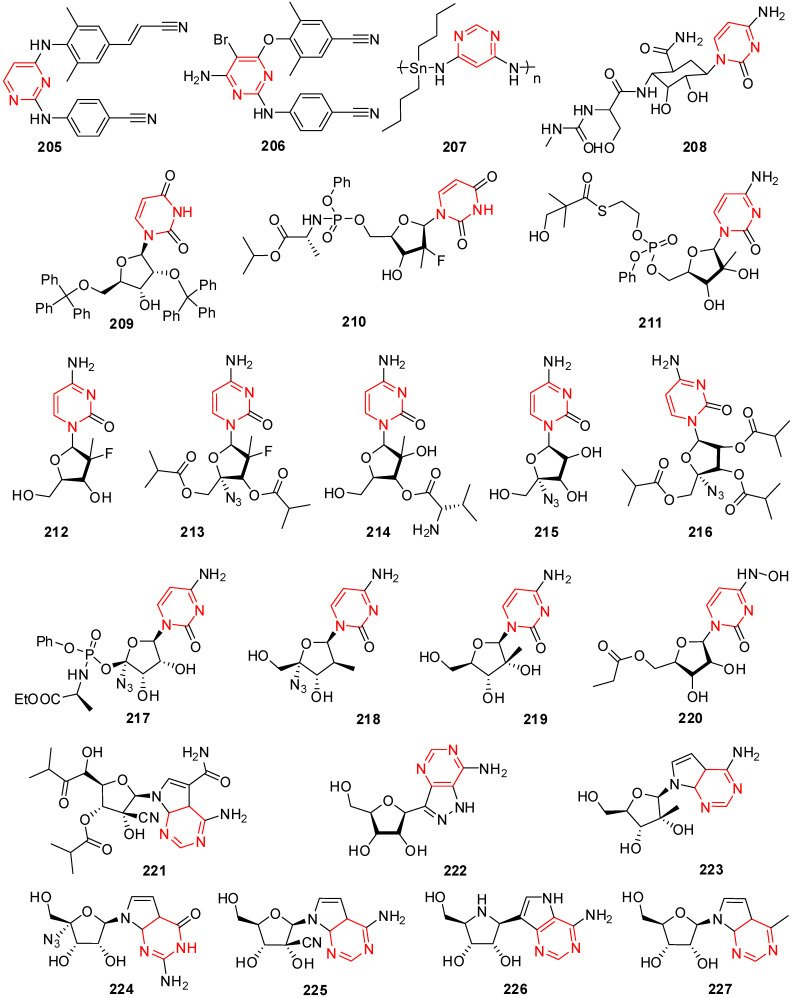
Pyrimidine derivatives with antiviral activity.

Two diaryl pyrimidine derivatives, rilpivirine (**205**) [277] and etravirine (**206**) (Figure 12) [278,279], have been therapeutically authorized for the treatment of HIV infection. Various polymers were developed with acyclovir as the Lewis base, concentrating on materials with organotin for their antiviral properties [226]. The reaction of several 4,6-diaminopyrimidines with dibutyltin dichloride was used to examine organotin polyamines. Many of these polyamines showed some capacity to stop viral development. The 4,6-diaminopyrimidine (**207**) substituted compounds expressed in Figure 12, nucleoside derivatives, are most likely responsible for the antiviral action [280]. The most potent registered plant viral inhibitor, ningnanmycin (**208**) (Figure 12), demonstrated a 56% in vivo therapeutic efficacy at a concentration of 500 μg/mL. 2′, 5′-di-O-trityluridine (**209**) and 3′, 5′-di-O-trytiluridine were synthesized as antagonists of YFV and DENV-2 reproduction and showed excellent antiviral efficiency and promise for cytotoxicity in Vero cells [281,282,283]. 

Sofosbuvir (SOF) (**210**), a phosphoramidite prodrug of 2′-fluoro-2′-C-methyluridine (Figure 12) synthesized by Gilead Sciences, Inc., is one of the most influential and selective nucleotide polymerase inhibitors. As a non-structural 5B (NS5B) polymerase inhibitor for the treatment of persistent HCV infection, SOF was given FDA approval in December 2013 [284,285,286]. Sofosbuvir has a good safety profile and little mitochondrial toxicity since it performs poorly when used to detect human mitochondrial RdRp [287,288]. The in vitro action of 2′-C-methylated nucleosides towards ZIKV was described. For the subsequent testing of ZIKV infection treatment in rodent models, these compounds served as the basis for the structure-based optimization and logical design of effective prodrugs [289]. Computational studies described that there may be a steric conflict between the 2′-C-methyl group of the nucleotide inhibitor that is integrated and the endogenous nucleotide substrate [290].

Sofosbuvir and other 2′-C-methylated hybrids (Figure 12) containing a fluorine atom or a hydroxyl group at the 2′-position function as efficient chain terminators such as IDX-184 (**211**) [291,292], PSI-6130 (**212**) [293,294,295,296], Mercitabine (**213**) [297,298], Valopicitabine (**214**) [299,300], 4′-azidocytidine (R-1479) (**215**), and its prodrug Balapiravir (R-1626) (**216**) [301,302,303]. 4′-azidocytidine (R-1479) (**141**), a 4′-azido modified nucleoside analog, exhibited micro- and nanomolar in vitro antiviral action towards TBEV [236]. Balapiravir, on the other hand, has reportedly shown potent antiviral in vitro efficacy towards several DENV serotypes. For DENV illnesses, it was the first effective antiviral medication to receive clinical approval. In the past couple of years, numerous effective NS5B inhibitors have been described, like Valopicitabine (**140**) [304] and R-1626 (**142**) [305]. The viral RNA-dependent RNA polymerase (RdRp), which is essential for replicating the HCV RNA genome, is encoded by the non-structural protein 5B (NS5B) [306]. Although alternative targets are being studied, most novel molecular antagonist strategies to HCV should be directed toward inhibiting significant viral targets, including the NS5B RdRp (substituted to HIV RT) and the NS3-4A protease (substituted to HIV protease) [307,308].

Surprisingly, when tested against HCV, nucleoside derivatives like 2′-C-methyl-4′-azidocytidine failed to show any antiviral property; however, the related 5′-monophosphate prodrugs (**217**) (Figure 12) demonstrated noticeably improved virus inhibitory effects with EC values in the micromolar ranges and without apparent cytotoxicity [309]. 

The intriguing substance 4′-azido-2′-deoxy-2′-C-methylcytidine (**218**) (Figure 12) and its ester prodrugs were shown to have in vitro antiviral action [310]. Using an effective synthetic ZIKV RdRp, the triphosphate derivatives of 2′-C-methylated nucleosides demonstrated outstanding inhibition efficacy in an in vitro polymerase experiment [311]. 

Furthermore, even when given three days after infection, 2′-C-methylcytidine (**219**) (Figure 12) protected the hamsters given a fatal dose of YFV, and the nursing mice were tested for DENV [312].

Molnupiravir, also known as EIDD-2801 (**220**), is an antiviral drug still in clinical trials that presents good oral bioavailability and broad-spectrum anti-influenza efficacy (in in vitro and in vivo studies) [313]. It is a prodrug, being hydrolyzed in plasma to its active form, N^4^-Hydroxycytidine, which is a cytidine analog (Figure 12).

Chen and his colleagues designed and prepared the isobutyryl ester prodrug of C7 carbamoyl substituted NITD008, referred to as NITD-203 (**221**) (Figure 12). NITD203 magnificently exhibited a nanomolar anti-DENV effect and enhanced pharmacokinetic efficacy when dosed orally [238].

Formycin (also known as formycin A or 7-amino-3-(b-D-ribofuranosyl)-pyrazolo[4,3-d]) (**222**) (Figure 12) is a C-nucleoside antibiotic that was developed by the mold S. candidus [314]. It is an adenosine nucleoside isomer that occurs naturally with a pyrazole group and antiviral effects. It perfectly replaced the adenosine unit at the nucleotide level in various enzymatic processes [315].

After Zika virus epidemic breakouts in Latin America and Oceania, 7-Deaza-2′-C-methyladenosine (**223**) (Figure 12) was the initial ZIKV nucleoside-based inhibitor discovered and described in conjunction with other 2′-C-methylated compounds [289,316]. 7-Deaza-2′-C-methyladenosine showed anti-ZIKV activity on immortalized cell lines and induced pluripotent stem cell-derived neuronal cell types, motor neurons, including cortical neurons and astrocytes [317]. 5-aza-7-deazaguanosine (ZX-2401) (**224**), in combination with interferon, demonstrated synergistic in vitro anti-YFV efficacy (Figure 12) [318].

When evaluated in A549 cells, BHK-21 cells, Huh-7 hepatocarcinoma cells, and human peripheral blood mononuclear cells (PBMCs), the 7-deaza-2′-C-ethynyladenosine (NITD008) (**225**) in Figure 12 prevented DENV of different serotypes at a micromolar level, exhibiting a significantly formed cytotoxic activity with more than 100 mM of CC_50_ in comparison to 2′-C-ethynyladenosine [319]. Additionally tested for ZIKV, YFV, and WNV resistance, NITD008 showed exceptional in vitro inhibitory activity and a protective effect in mice efficacy models towards Zika virus and WNV infections [319,320]. NITD008 was also utilized to efficiently prevent the in vitro reproduction of POWV, TBEV, KDFV, AHFV, and OHFV with low micromolar or nanomolar levels detected in numerous methods for screening cells [321]. 

The BCX4430 (**226**), an imino-C-nucleoside, in Figure 12 was first referred to as an inhibitor of filovirus infection, exhibiting antiviral activity against members of RNA viruses, particularly Picornaviridae, Arenaviridae, Bunyaviridae, Orthomyxoviridae, Flaviviridae, Coronaviridae, and Paramyxoviridae families [322]. The effective treatment of BCX4430 is used to treat Ebola virus disorders and showed impressive outcomes in in vitro tests. The mechanism of action of BXC4430 involves its uptake by pathogenic RNA polymers and prevents the replication of RNA. In vitro *studies* presented the potential effects of the Ebola virus [322,323,324]. The mosquito-transmitted flavivirus BCX4430 is effective against a variety of flaviviruses, including WNV with an EC_50_ of 2.33 mM [325], and participants of the ZIKV’s African and Asian lineages (3.8–11.7 mg/mL) [326].

6-methyl-7-deazaadenosine (**227**), a hydrophobic analog of adenosine (Figure 12), demonstrated nanomolar antiviral efficacy against DENV-2 in both a luciferase-driven DENV-2 replicon test and a Vero cell-based screening approach. No cytotoxicity was seen after 7 h of dosing [327].

## 16. Triazine Derivatives

**Figure 13:**
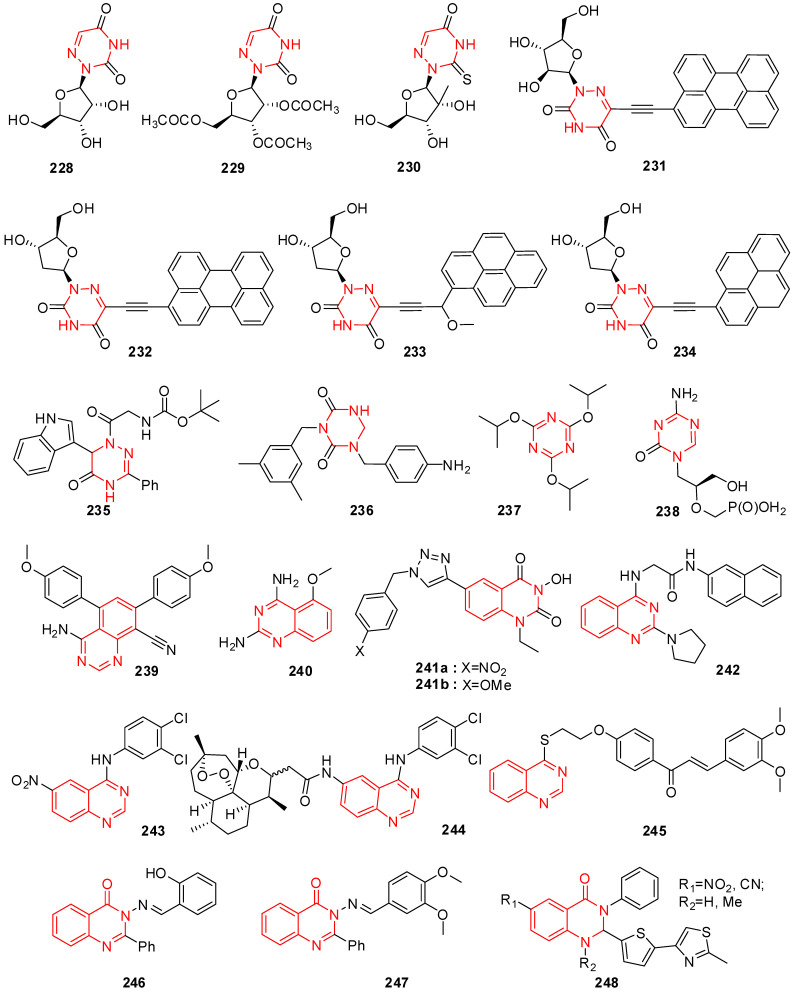
Triazine, Quinazoline, and Quinazolin-ones derivatives with antiviral activity.

Triazines are an important class of nitrogen heterocycles that display diverse biological activities. They form an integral part of different therapeutically interesting compounds due to their similarity to the biologically active purine and pyrimidine scaffolds [116]. Orotidine monophosphate decarboxylase is inhibited by 6-azauridine (**228**) and its analogs (Figure 13), preventing the cell’s de novo pyrimidine production [142,328]. 6-Azauridine was found to be effective against several flaviviruses carried by arachnids [329]. Meanwhile, it showed only minor cytotoxicity and delayed the development of the host cells [330]. Minimal micromolar efficacy towards AHFV and WNV was found in an in vitro triacetate prodrug of 6-azauridine (**229**) [329], and extremely low toxicity was found in both animal and human trials. Another analog, 2-thio-6 azauridine (**230**), has a slightly inhibitive impact on WNV [328]. 

Excellent inhibitors of TBEV were identified as 5-(Perylen-3-yl)ethynyl-arabinouridine (**231**) and 5-(perylen-3-yl)ethynyl-2′-deoxyuridine (**232**) [331]. The firm ethynyl linker and the perylene component were determined to be antagonistic for the minimal cytotoxicity at concentrations greater than 50 mM and nanomolar anti-TBEV potency. Unexpectedly, the anti-TBEV potency of uracil nucleosides with a pyrene moiety, such as 5-[(pyren-3-yl)methoxypropyn-1-yl]-2′-deoxyuridine (**233**) and 5-[(pyren-1-yl)ethynyl]-2′-deoxyuridine (**234**), was almost 10-fold lower. Even if they are not employed as medicines, these substances might aid in our knowledge of the various nucleoside scaffolds’ diverse mechanisms of action (Figure 13) [331].

A series of 1-acyl-6-indolyl-3-phenyl-1,6-dihydro-1,2,4-triazin-5(4*H*)-ones were synthesized and the cytotoxic and antiviral actions of the 12 resulting compounds were studied using the vaccinia virus. The cytotoxic and antiviral activities of the resulting compounds were tested using the vaccinia virus. Most of the compounds were found not to be cytotoxic. Indolyl 1,2,3-triazine derivatives, especially compound **235**, efficiently inhibited the reproduction of the vaccinia virus [332]. 

New series of triazine analogs were synthesized and determined for anti-HIV-1 activities based on the inhibition of virus-induced cytopathogenicity in MT-4 cells. The cytotoxicity of the compounds was evaluated by assessing the viability of mock-infected cells. Results revealed that a dihydro-1-(4-aminobenzyl)triazine analog (**236**) showed satisfactory anti-HIV-1 activity with an EC_50_ of 0.110 mM and a selectivity index (SI) of 909. Furthermore, molecular modeling analyses were performed to explore the major interactions between HIV-1 RT and potent inhibitors [333].

Newly designed 2,4,6-trisubstituted symmetrical 1,3,5-triazine (TAZ) derivatives were synthesized and tested for biological evaluation. Among the tested trisubstituted TAZ derivatives, some *C*Ssymmetrical alkoxy-amino-substituted TAZ derivatives showed significant antiviral activity against herpes simplex virus type-1 (HSV-1). The compound with the highest level of antiviral activity was *C*3-symmetrical trialkoxy-TAZ derivative **237** (Figure 13), which showed a considerably high selectivity index (IC_50_/EC_50_ = 256.6) [334].

Among the 5-azacytosine congeners of acyclic nucleoside phosphonate analogs, compound **238** (Figure 13) showed potent and selective activity against several DNA viruses, including different herpesviruses (HSV-1, HSV-2, VZV, HCMV, and HHV-6), adenovirus (Ad2), and poxvirus (vaccinia virus) with EC_50_ values of 0.71 µg/mL for Ad2, 2.56 µg/mL for the vaccinia virus, and 0.02–0.6 µg/mL for herpes viruses. Its activity was comparable to that of the reference drug (S)-HPMPC against HSV-1, HSV-2, and the vaccinia virus, or 2–7-fold more active against VZV, HCMV, HHV-6, and Ad2. This compound was proved to be 2-fold less cytotoxic for HEL cells than (S)-HPMPC with a CC_50_ value of 140 µg/mL compared to 61 µg/mL for (S)-HPMPC. For all these DNA viruses, compound **238** showed a 2–16-fold higher antiviral selectivity index (ratio of CC_50_ to EC_50_) than (S)-HPMPC [335].

## 17. Quinazoline/Quinazolin-ones Derivatives

Quinazoline molecules are well documented in drugs and medicine. They are crucial components of various natural and manufactured medications with antiviral action against certain viruses [336,337,338,339,340,341]. Non-fluorescent quinazolines are found in various clinically used medicines with four different substitution patterns. The production of luminous 4,5,7,8-substituted quinazolines with EC_50_ values as low as 0.6 ± 0.1 mM over human cytomegalovirus (HCMV) was developed by Held et al. Additionally, the production of artesunic acid-quinazoline compounds and their effectiveness towards HCMV were proven with EC_50_ values as low as 0.1 ± 0.0 mM. The novel quinazolines show a high level of action against HCMV throughout time. With a reduced in vitro EC_50_ value than the standard, such as ganciclovir, the hybrid **239** (Figure 13) demonstrated the greatest antiviral efficacy [342].

Recently, several 2,4-diaminoquinazoline analogs like **240** (Figure 13) and other antiviral drugs with strong inhibitory properties have been shown [343]. (4-Nitrophenyl)-substituted quinazoline derivative **241a** shown in Figure 13 outperforms cidofovir (EC_50_ = 25 μM) in activity against vaccinia virus 15 times with an EC_50_ of 1.7 μM and SI > 58.8, while 4-methoxy substituted quinazoline derivative **241b** outperforms zalcitabine (EC_50_ = 7.2 μM) against type-2 adenovirus with an EC_50_ of 6.2 μM and SI > 16.1 [344].

Quinazolines derivatives were analyzed against influenza A/WSN/33 (H1N1) virus and the structure–activity relationship revealed that the best results were shown by derivative **242** (Figure 13) which was superior in activity with an EC_50_ of 1.29 ± 0.01 μM to ribavirin (EC_50_ = 15.36 ± 0.93). It was noted that derivatives of *S*-acetamides are inferior in activity to the corresponding *N*-acetamide derivatives [345].

Several 4-arylaminoquinazolines **243** effectively suppress the replication of human cytomegalovirus with an EC_50_ value of 0.05 ± 0.02 μM. Conjugates of 4-arylaminoquinazolines with the sesquiterpene lactone artemisinin exhibited antimalarial action. It was shown that derivative **244** is superior in anti-cytomegalovirus activity to ganciclovir with an EC_50_ of 0.15 ± 0.05 μM [336].

2-Sulfanylquinazolines **245** containing the chalcone fragment (EC_50_ 138.1 μg/mL) are superior to ribavirin (EC_50_ 436.0 μg/mL) in activity against tobacco mosaic virus [346].

A class of 3-(benzylideneamino)-2-benzylquinazoline-4(3*H*)-one substituents represented in Figure 13 was developed and assessed for antiviral action against the herpes simplex virus-1 TK-KOS ACVr, Punta Toro virus, para influenza-3 virus, herpes simplex virus-1 (G), herpes simplex virus-1 (KOS), influenza A H1N1 subtype, Sindbis virus, reovirus-1, respiratory syncytial virus, influenza B, feline coronavirus (FIPV), and feline herpes virus influenza A H3N2 subtype. Conversely, molecules with 2-hydroxy substitution (**246**) showed more significant antiviral activity [341]. In Vero cell cultures, it was discovered that compound **247** prevented the replication of viruses such as reovirus-1, Punta Toro virus, Coxsackie virus B4, para influenza-3 virus, and Sindbis virus [347]. 2-(Thiophen-2-yl)-2,3-dihydroquinazolin-4(1*H*)-ones **248** (Figure 13) demonstrated high activity against human cytomegalovirus and polyomavirus [348].

## 18. Pyrazine Derivatives

**Figure 14:**
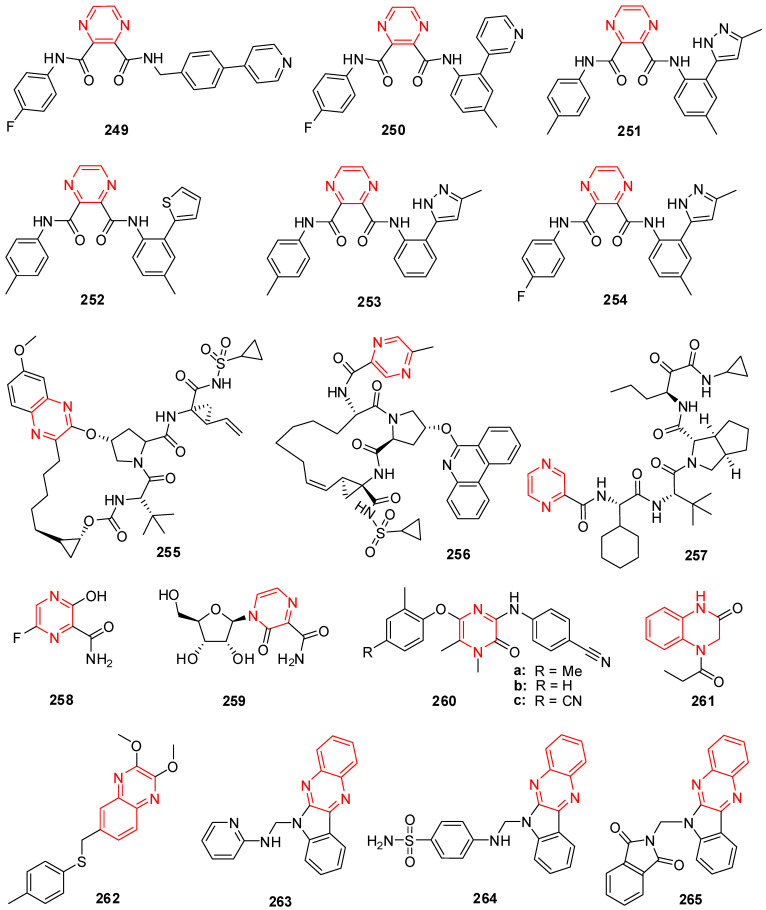
Pyrazine and Quinoxaline derivatives with antiviral activity.

Saudi et al. synthesized a model class of pyrazine 2,3-dicarboxamides (P23DCs) and identified them based on their antiviral in vitro efficacy towards the yellow fever virus (YFV) and dengue virus (DENV). Molecules **249** and **250** (Figure 14) in this series significantly reduced the replication of DENV in Vero cells (EC_50_ = 0.93 μM) [86].

Numerous pyrazine-2,3-dicarboxamide and phthalic diamide derivatives were produced and SAR studies were conducted by Saudi et al. These compounds were then tested in cell-based studies for antiviral efficacy and cytotoxicity towards the yellow fever virus (YFV) and dengue virus (DENV). DENV replication was prevented by 14 substances (EC_50_ = 0.5–3.4 mM), including compounds **251**–**254**, which showed potent inhibition with an EC_50_ of 0.5 mM and selectivity indices (SIs) greater than 235. Seven compounds also showed promising action against the YFV (EC_50_ = 0.4–3.3 mM), with molecule **253** being the most successful with an EC_50_ value of 0.4 mM and an index of the selectivity of more than 34 [349].

Due to the rapid emergence of hepatitis C virus (HCV) drug resistance, Shah et al. reported the synthesis of quinoline derivatives as HCV drug candidates to target the NS3/4a protease, which plays crucial roles in processing viral protein and replicating viral RNA [350]. It also inhibits the production of interferons that can enhance the immune system against viral infection [351]. The new scaffold was developed based on previous clinical candidate MK-5172 (**255**) with a quinoline moiety instead of quinoxaline.

ABT-450 (**256**) (Figure 14) has EC_50_ values for stable HCV replicons from genotypes and is an effective antagonist of the HCV NS3/4A protease 1b, 1a, 2a, 4a, 3a, and 6a of 1.0, 0.21, 5.3, 19, 0.09, and 0.69 nM, respectively. The sites 155, 156, and 168 in NS3 of genotype-1 were the most often encountered amino acid variations by ABT-450 in vitro, and genotype-1a and genotype-1b replicons (219- and 337-fold, respectively) have the maximum level of ABT-450 tolerance when the D168Y mutation is present. In a 3-day monotherapy trial with patients with HCV genotype 1, ABT-450 was coadministered with ritonavir, a cytochrome P450 3A4 inhibitor that has previously been demonstrated to significantly increase in peak, trough, and total drug exposures of ABT-450. After the 3-day dosing period, a mean highest HCV RNA decline of 4.02 log10 was seen across all doses. D168V and R155K in genotype-1a and D168V in genotype-1b were the most frequently preferred variants in these patients. However, compared to lesser dosages, the choice of resistant variants was dramatically decreased at the maximum ABT-450 dose [352].

Telaprevir (**257**), with the IUPAC name (1*S*,3a*R*,6a*S*)-2-[(2*S*)-2-[[(2*S*)-2-Cyclohexyl-2-(pyrazine-2-carbonylamino)acetyl]amino]-3,3-dimethylbutanoyl]-*N*-[(3*S*)-1-(cyclopropylamino)-1,2-dioxohexan-3-yl]-3,3a,4,5,6,6a-hexahydro-1*H*cyclopenta[c]pyrrole-1-carboxamide (Figure 14), is used for hepatitis C treatment, which was developed by Revill et al. [353]. In 2011, the US FDA authorized the use of telaprevir with pegylated and ribavirin interferon [184,185]. Currently, the main methods of preventing influenza are vaccination and antiviral medications. The anti-influenza medication RNA-dependent RNA polymerase (RdRp) is one option of favipiravir (**258**) (Figure 14) [354].

T-1106 (**259**) (Figure 14), a pyrazine analog hybrid that has been ribosylated, shows inhibition towards the HCV RdRp in enzyme studies [355]. T-1106 and ribavirin produced better results than a single one [356].

Heeres et al. have developed a pyrazinone-based non-nucleoside HIV-1 reverse transcriptase (NNRT) inhibitor [357]. A cyano substituent at the aniline’s 4-position was discovered to be the more incredible option for the optimal action. Among these analogs, the most effective compounds for the wild-type HIV-1 LAI virus were molecules **260a** and **260b** (Figure 14), with 0.003 μM and 0.004 μM activity, respectively.

Furthermore, several single and double mutant virus strains were used to assess the described compounds. When compared to efavirenz, molecule **260c** showed reduced effectiveness against wild-type HIV-1 LAI and a single Y181C mutation (Figure 14). Molecule **260c**, with the activity of 0.168 and >10 μM, was more effective than efavirenz against double L1001 + K103N viral mutant strains.

## 19. Quinoxaline Derivatives

The 3DQSAR study allowed for the structure to be optimized followed by the synthesis of quinoxaline analogs (Figure 14) which showed high antiviral activity against wild and mutant (K103N) HIV reverse transcriptase. Compound **261** showed a higher EC_50_ value of 3.1 nM and an SI value of 3.18 compared to the commercial drug nevirapine [358].

Quinoxaline derivatives were identified to have low toxicity and high activity with EC_50_ values of 0.06–3.8 μM against Coxsackie B5 (CV-B5) enterovirus (Figure 14). Structure–activity analysis shows that for the selective activity to be manifested, the carboxyl group in compounds must be located in the *para* position of the benzene or pyridyl fragment for the sulfanyl group. Compound **262** which showed the highest activity against the CV-B5 virus (EC_50_ 0.09 ± 0.01 μM) was chosen as the lead compound [359].

The antiviral effectiveness of indophenazine compounds towards HSV [360] and suppression of viral DNA and RNA synthesis was evaluated [361]. Through the Mannich reaction, a class of nitrogen-containing moieties, indophenazine, was developed, and their antiviral effectiveness towards a range of human viral diseases was tested. The synthesized compounds were tested for antiviral efficacy towards the vaccinia viruses and HSV-1,2 in HEL cells. Vaccinia virus and herpes simplex virus-1 were suppressed by the molecules 10*H*-indolo-2-Amino pyridine [3,2-b] quinoxalines (IP-2AMP) (**263**) and 4-Aminobenzene sulfonamide-10*H*-indolo [3,2-b] quinoxalines (IP-SN) (**264**) represented in Figure 14 at doses of 12 g/mL and 100 g/mL, respectively. Additionally, using zidovudine (AZT) as a standard, these molecules were screened for in vitro antiviral efficacy against HIV-2 (ROD) and HIV-1 (IIIB) replication in MT-4 cells. In MT-4 cells, the phthalimide analog suppressed HIV-2 replication with EC_50_ values of 11.60 μg/mL and CC_50_ values of 61.63 μg/mL [362].

The newly created indophenazine analog, indolo [2,3-b]quinoxalin-6-ylmethyl-isoindole-1,3-dione (IP-PTH) (**265**) represented in Figure 14, prevented HIV-2 replication in MT-4 cells.

## 20. Piperazine Derivatives

**Figure 15:**
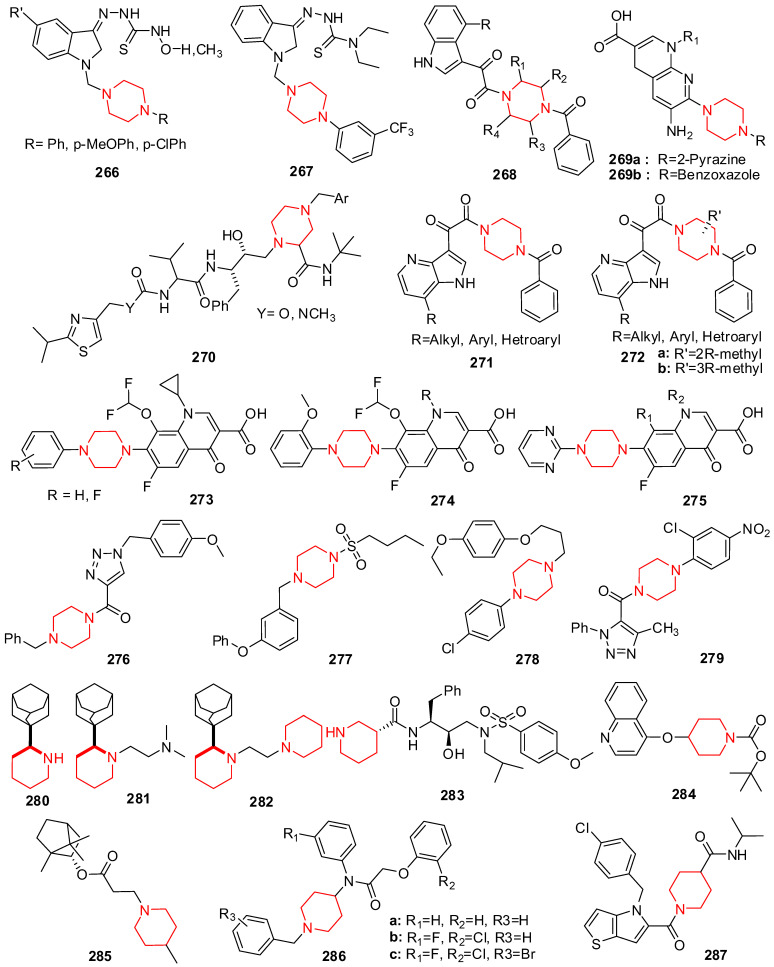
Piperazine and Piperidine derivatives with antiviral activity.

Recently, emphasis has been placed on the development of bis(heteroaryl)piperazine analogs as NNRTIs for the treatment of HIV-1 [363]. This work has also revealed information on structure–activity correlations, which has sparked more thought about how to build methods for future treatments for HIV. A synthetic isatinyl thiosemicarbazones analog, like compound **266** (Figure 15), with piperazine derivatives has been produced, created, and evaluated for its effectiveness in the HIV-1 IIIB strain to treat HIV-TB co-infection. Intense action against the reproduction of HIV-1 cells was produced by several compounds [364]. 

A compound with a 1-(3-trifluorometheylphenyl) piperazine moiety delivered excellent action (EC_50_ = 2.62 μM and SI = 17.14) with some cytotoxicity, but not much, and some molecules demonstrated significant action below their toxic effects. Thiosemicarbazone derivatives based on isatin, like **267** (Figure 15), were created and investigated for in vitro anti-HIV efficacy towards the strain HTLV-IIIB [43]. 

The impact of piperazine-substituted analog **268** (Figure 15) on viral HIV-1 adhesion to the host cell receptor has recently been studied in indole-based substituents. These investigations demonstrated the piperazine ring as a crucial pharmacophore element that prevented HIV-1 attachment, positioning the benzamide and indole glyoxamide in a topological relationship that complimented the binding site on gp120 [365], where, R_1_, R_2_, R_3_, R_4_ = H, CH_3_, (R)CH_3_, (S)CH_3_, Ph, (S)PhCH_2_, CH_3_CH_2_, CH_3_CH_2_CH_2_, CH_3_(CH_2_)_4_, (CH_3_)_2_CH, (CH_3_)_3_C, CONH_2_, (S)CO_2_CH_3_, COOH, CO_2_CH_2_CH_3_, CH_2_OH, cis-CH_3_, trans-CH_3_, etc.

By trying structural alteration on 6-aminoquinolone analogs, several authors developed novel compounds with piperazine derivatives. As NNRTIs towards MT-4, PBMCs, and CEM cell lines, new analogs were evaluated for their in vitro anti-HIV action against HIV-1 (IIIB) and HIV-2 (ROD) strains. Analogs **269a** and **269b** (Figure 15) were discovered to be highly efficient, with an IC_50_ of 0.0087–0.7 μg/mL and an exemplary extent of therapeutic efficacy at greater than 1000 on CEM cell lines [366]. 

Chen et al. developed the inhibitors of the structure of Piperazine hydroxyethylamine substituents **270** (Figure 15) and they displayed up to 20 times higher anti-HIV activity than ritonavir in the presence of human serum. Piperazine hydroxyethylamine derivatives (**270**) inhibited the HIV protease enzyme, and it was noted that 25 derivatives exhibited an IC_50_ of less than 0.3 μM with low cytotoxicity, which is more efficient than the medication Ritonavit having an IC_50_ of 0.81 μM [367].

To identify oral HIV-1 adhesion antagonists, a series of 4-azaindole derivatives expressed in Figure 15, including unsubstituted benzoyl piperazine (**271**), 2*R*-methyl benzoyl piperazine (**272a**), and 3R-methyl benzoyl piperazine (**272b**), was developed and tested. Most analogs showed less than 1 nM of EC_50_ and significantly reduced cytotoxicity. When compared to counterparts viewing unsubstituted benzoyl piperazine, the substitution of 2*R*-methyl benzoyl piperazine (**272a**) or 3*R*-methyl benzoyl piperazine (**272b**) was necessary to create the most potent action (**271**) [164].

Many arylpiperazinyl fluoroquinolones were developed and are now being used as novel anti-HIV medications. It was shown that for the piperazine ring to demonstrate potential activity against cells with persistent infections, the replacement of aryl moieties for the nitrogen atom was essential. Numerous compounds containing piperazine derivatives (Figure 15) had outstanding levels of cytotoxicity and an IC_50_ of 0.059–0.97 μM, but derivatives containing the 1-(2-pyridyl) and 1-(2-pyrimidyl) piperazine rings (**273**, R = H) displayed IC_50_ values of 0.25 μM and 0.97 μM, respectively. The IC_50_ levels for the compounds 1-(2-methoxyphenyl)piperazine ring (**274**) and 1-(2-pyrimidyl)piperazine ring (**275**) were 0.094–5.6 μM and 0.14–0.97 μM, respectively. Additionally, the most robust hybrid in this series has piperazine ring systems with a benzyl or fluorophenyl (**273**) group [368].

Structurally diverse piperazine analogs were discovered and tested for their outstanding antinorovirus action. Derivatives **276** and **277** (Figure 15) were found to be the best antagonists of norovirus, with ED_50_ levels of 7.2 and 6.7 μM, low cytotoxicity, and therapeutic indices of 155.4 and 121.2, respectively [369].

Synthetic chloro-pyridazine piperazines have been produced and biologically examined; they are HRV-3 inhibitors. The initial screening showed the influential activity of the said derivatives against HRV-3 with an EC_50_ level at 3.1–25 ng/mL, a higher therapeutic index of 156–4876, and low cytotoxicity. The most effective compound **278** (Figure 15), which bonded the functionality of the ethoxy group to a benzoyl ring linked to a piperazine structure, displayed an EC_50_ level of less than 3.2 ng/mL and a therapeutic index of more than 4875 [370]. 

Additionally, studies have been conducted to determine the effectiveness of piperazino-1*H*-1,2,3-triazole-4-carboxamide analogs against the influenza A virus. Influenza A/HK/8/68 (H3N2) and influenza A/WSN/33 (H1N1) viral stains were used in the bioassay. Several analogs showed minimum MICs of 1.08–16.16 μM against the H_3_N_2_ strain and an IC_50_ value of 0.32–16.98 μM against the H1N1 strain with the least amount of cytotoxicity (CC_50_: >100 μM). The most potent derivative **279** mentioned in Figure 15, which has Cl and NO_2_ groups at the *ortho* and *para* positions of the phenyl ring of the piperazine moiety, has IC_50_ values of 0.68 μM and 1.97 μM against HINI and H3N2 strains, respectively, and has a cytotoxicity of more than 100 μM [371].

## 21. Piperidine Derivatives

The anti-influenza drugs Amantadine and Rimantadine are medications that, at micromolar quantities, prevent virus reproduction [372,373,374]. Since 2008, many aminoadamantane carbocycles and heteroatoms with anti-influenza A virus activity have been produced in laboratories [374,375,376]. The influenza A and B viruses and human immunodeficiency virus types-1 and 2 (HIV-1) were tested for their antiviral efficacy by Stamatiou et al., who also produced 2-(1-adamantyl)hexahydroazepines and 2-(1-adamantyl)piperidines. The influence of ring size on anti-influenza A action was investigated. The hexahydroazepine analog was inactive. However, the rimantadine analog **280** (Figure 15) was four times as powerful as the medication rimantadine. Thus, the influenza A vaccine activity was significantly reduced after the growth of a five-membered (pyrrolidine) or six-membered (piperidine) heterocyclic ring to a seven-membered (hexahydroazepine) heterocyclic ring. The effective molecules **281** and **282** (Figure 15) were synthesized by replacing the dialkyaminoethyl group on piperidine 10; **281** was effective towards influenza A virus, and **282** was vital towards HIV-1 [186].

Zhu et al. designed a novel class of HIV-1 protease inhibitors with flexible piperidine as the P2 ligand and they exhibited good-to-excellent inhibitory effects on enzymatic activity and viral infectivity. In particular, inhibitor **283** with (R)-piperidine-3-carboxamide as the P2 ligand showed an enzyme Ki value of 29 pM and antiviral IC_50_ value of 0.13 nM, more than a six-fold enhancement of activity compared to DRV. Moreover, inhibitor 3a exhibited potent antiviral activity against subtype C variants with low nanomole EC_50_ values [377]. 

Guoxin et al. prepared a series of different analogs that were proven to have an inhibitory effect against the influenza virus. Structure–activity relationship studies indicated that the ether linkage between quinoline and piperidine is crucial to the inhibitory effect. Compound **284** showed a superb inhibitory effect on a diversity of influenza virus strains and showed a good ability to interfere with the early-to-middle stages of influenza virus replication [378].

No antiviral medication is licensed to treat Marburg virus illness (MVD). Using pseudotype systems, Kononova et al. screened a vast array of natural chemicals for their ability to block the entry of viruses. The most robust antiviral activity was in bornyl ester analogs of saturated nitrogen-containing compounds. Borneol is harmless and inactive against both pseudotypes, while its analogs showed different degrees of toxicity and antiviral efficacy. Six low-toxic borneol analogs were solidified as particular MarV-GP-mediated infection inhibitors with SC > 10. The most virus-specific activity was shown by molecule **285** (Figure 15) with a methylpiperidine moiety, which was twice as significant as the reference [379].

Rokhyatou et al. designed and prepared novel 1,4 disubstituted piperidine derivatives that were tested for their activity against chloroquine-sensitive and chloroquine-resistant strains of *P. falciparum*. Compounds **286a**–**c** were the most potent compounds in the prepared library [380].

Guo et al. evaluated the in vitro activities of a piperidine-4-carboxamide compound (**287**) against human α-coronavirus NL63, β-coronaviruses OC43, and the alpha and delta variants of SARS-CoV-2 in several cell lines. **287** (Figure 15) showed antiviral activity in NL63-infected Vero and MK2 cells with EC_50_ values of 2.5 ± 0.15 μM and 1.5 ± 0.2 μM, respectively. The cellular toxicity in both cell types was > 300 μM. The EC_50_ of **287** in OC43-infected human foreskin fibroblasts was 1.5 ± 0.01 μM. **287** inhibited SARS-CoV-2 in both Vero E6 and Calu-3 cells [381].

## 22. Pyridine Derivatives

**Figure 16:**
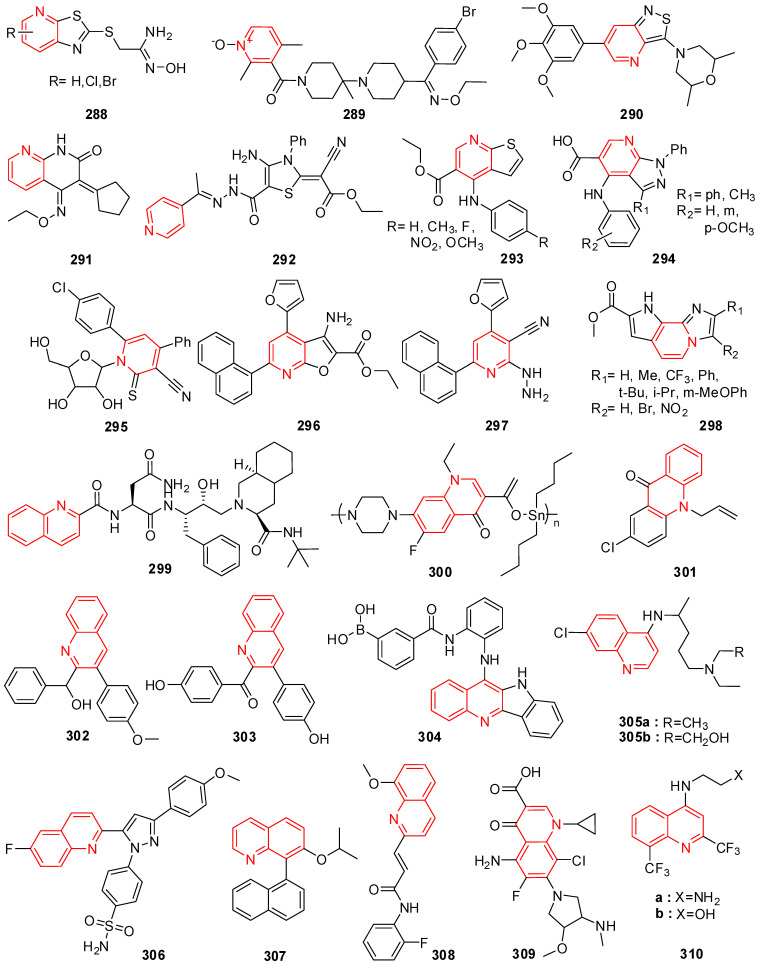
Pyridine and Quinoline derivatives with antiviral activity.

The abundance of pyridine in vital supplements like niacin and pyridoxine (vitamin B6) and highly poisonous alkaloids like nicotine provides more proof of the compound’s powerful biological activity. Over 7000 medications currently used in the pharmaceutical business have pyridine as their core component. Thiazolo[5,4-*b*]pyridine (**288**) Oxime analogs have shown activity against the influenza B-Mass virus, according to research by Abele et al. The most effective HIV inhibitors are pyridine (**289**) and naphthiridine (**291**) oxime analogs (Figure 16) [382]. 

Isothiazolo[4,3-*b*]pyridines (**290**) (Figure 16) were synthesized by Martinez-Gualda et al. and screened for antiviral efficacy as cyclin G-associated kinase inhibitors. With different substitutions of pyridine, these molecules were established to exhibit good activity against the dengue virus [383].

El-Hawash et al. assessed the potential of the compound hydarzone of 3- and 4-acetyl pyridine (**292**) to prevent the proliferation of HCV in both RNA(−) and RNA(+) strains as well as to have antitumor action (Figure 16) [384]. Ruthenium-complexed bipyridinyl compounds exhibit antiviral action towards the hepatitis C virus (HCV) [385].

Herpes simplex virus type-1 (HSV-1) was effectively inhibited by novel 4-(phenylamino)thieno[2,3-b]pyridine analogs (**293**) that were developed by Bernardino et al. Their structure–activity relationships (SARs) and those of 4-(phenylamino)-1*H*-pyrazolo [3,4-b]pyridine analogs (**294**) demonstrate a variety of biological activities, including anti-HIV-1 and vaccinia virus activities (Figure 16) [386]. To treat HIV infections, Attia et al. developed various pyridine ribosides. The most effective anti-HIV medications were discovered to be 1-(-D-ribofuranosyl)-pyridine-2-thione (**295**), given in Figure 16 [387].

The foundational element for the formation of several fused heterocyclic compounds, such as furopyridine, pyridotriazine, and pyridothiadiazepinthione, as well as non-fused heteroatom compounds, such as pyridin-2-yl-1*H*-pyrazole, was produced by Salem et al. using a straightforward four-component one-pot reaction. It was discovered that the new chemicals were antiviral substances [388].

Adenovirus type-7 and the rotavirus Wa strain were both susceptible to the therapeutic effects of molecules **296** and **297** (Figure 16). With adenovirus type-7 and the rotavirus Wa strain, molecule **297** showed 50% and 53.3% reductions, respectively. With adenovirus type-7 and the rotavirus Wa strain, molecule **296** demonstrated 53.3% and 60% viral intensity reductions, respectively. The genome of the rotavirus Wa strain may have a more significant reduction efficacy than the adenovirus type-7 strain. It might indicate that molecules **296** and **297** might significantly impact DNA viruses more than RNA viruses. 

A family of imidazo [1,2-a]pyrrolo [2,3-c] pyridines (**298**) represented in Figure 16 were discovered by Chezal et al. and are effective in the Pestivirus genus, the classical swine fever virus (CSFV), and border disease virus (BDV). A significant aspect of animal diseases caused by pestiviruses includes bovine viral diarrhea in cattle, classical swine fever in pigs, and border disease in sheep [389].

## 23. Quinolines Derivatives

Saquinavir (**299**), represented in Figure 16, is an HIV protease inhibitor used together with ritonavir (Norvir) and other medicines to cure human immunodeficiency virus (HIV) diseases [390]. The organotin polymers of norfloxacin (**300**) showed the inhibition of Epstein-Barr virus (EBV), varicella-zoster virus (VZV), and the cytomegalovirus (CMV), within the range of 2 mg/mL [113].

In a recent analysis, Mazzucco et al. identified several *N*-allyl acridones as strong viral inhibitors. The most potent DENV-2 inhibitors were also characterized for their antiviral efficacy and their mechanism of action, which targets viral RNA replication. The compound 10-allyl-7-chloro-9(10*H*)-acridone (**301**) in Figure 16 was effective in preventing the four DENV serotypes from infecting Vero cells in vitro, with an EC_50_ value of 12.5–27.1 μM. Additionally, the chemical is powerful in human HeLa cells. At doses up to 1000μM, there was no evidence of cytotoxicity. Mechanistic investigations showed that real-time RT-PCR measurements of viral RNA synthesis strongly indicated that viral entrance into the human host was unaffected [391].

Tseng et al. developed a brand-new sequence of 2-aroyl-3-arylquinoline compounds and tested them for anti-dengue virus activity. The DENV2 RNA expression in Huh-7-DV-Fluc cells was strongly inhibited by 2-(hydroxyphenylmethyl)-3-(4-methoxyphenyl)quinoline (**302**) and 2-(4-hydroxybenzoyl)-3-(4-hydroxyphenyl)quinoline (**303**), both of which had potencies that were roughly equivalent to those of ribavirin (Figure 16). There was a dose-dependent inhibition. At a dose of 100 μM, neither substance significantly damaged Huh-7-DV-Fluc cells, but it did prevent DENV replication in terms of viral protein and mRNA levels. However, the positive ribavirin test showed apparent cytotoxicity [392].

Wang et al. reported the synthesis of indoloquinoline derivatives as potential anti-influenza A agents to counter the drug resistance of viral strains. Compound **304** (Figure 16) exhibited superior properties compared to other derivatives in this scaffold with low cytotoxicity. This compound could interfere with cellular signaling pathways that are essential for viral replication and improve the survival rate of the mouse model in a histopathology study [393].

Chloroquine (**305a**), an antimalarial drug, exhibited antiviral activity against COVID-19 infection in vitro and showed its effectiveness in blocking virus infection at an EC_50_ of 1.13 μM concentration with a high selectivity index (SI). It could effectively inhibit viral infection at a low concentration and with good cytotoxicity compared to other FDA-approved antiviral agents. Additionally, it also effectively reduced viral copies by 10 μM 48 h post-infection in the nucleoprotein through immunofluorescence assays [394]. Hydroxychloroquine (**305b**), a chloroquine derivative, was found to have fewer adverse effects than chloroquine. Chloroquine **305a** (Figure 16), a quinolone analog typically used to treat malaria, has revealed antiviral effects against some viral infections. Of particular interest to human pathology is the inhibition of the hepatitis A virus [395,396,397].

Diarylpyrazolyl-substituted quinoline **306** (Figure 16), which exhibited higher inhibitory activity against DENV-2 (IC_50_ 0.81 μM, SI > 246.91) compared with ribavirin (IC_50_ 12.61 μM, SI 4.47), was considered as a potential antiviral drug against DENV. This compound also effectively inhibits other serotypes of DENV and reduces the clinical manifestations of the disease and mortality in mice infected with DENV [398].

Overacker et al. synthesized quinoline derivatives to target HIV RNase H [399]. Compound **307** (Figure 16) required a low concentration for antiviral activity and a high concentration for cytotoxicity in the in vitro infectivity assay with pseudoviruses. This compound also had a high selectivity index to inhibit the growth of HIV. For the mode of action, the compound showed better inhibition against the HIV-1 RNase H enzyme compared to weak inhibition (>100 μM) in HIV-1 integrase and HIV-1 RT.

Compound **308** (Figure 16) showed the highest in vitro activity against HIV-1 strains HIV-1VB59 and HIV-1UG070 in TZM-bl cell lines with IC_50_ values of 3.35 ± 0.87 and 2.57 ± 0.71 μM, respectively. Its ability to inhibit entry into the target cell with an IC_50_ of 1.40 ± 0.28 μM and therapeutic index (TI) of 38.29 as well as the process of fusion with the target cell with an IC_50_ of 0.96 ± 0.28 μM and TI of 55.83 are given as the purported mechanisms of action [400].

Quinolone analogs were screened by using BHK-21 cells infected with dengue virus type-2 (DENV-2), revealing their inhibiting capability of DENV-2 replication. For the lead compound **309**, the EC_50_ for the DENV-2 is 3.9 μM [401]; however, the antiviral mechanism of action of this compound is not entirely clear and requires further research.

Barbosa et al. have developed quinoline derivatives to inhibit the replication of arboviruses such as Zika (ZIKV) and chikungunya (CHIKV). 2,8-Bis(trifluoromethyl)quinoline derivatives **310a** and **310b** (Figure 16) showed the highest anti-ZIKV activity (EC_50_ = 0.8 μM), which is five times more effective than mefloquine, an antiviral drug approved by the Food and Drug Administration (FDA) [402].

## 24. Miscellaneous *N*-Heterocyclic Derivatives

**Figure 17:**
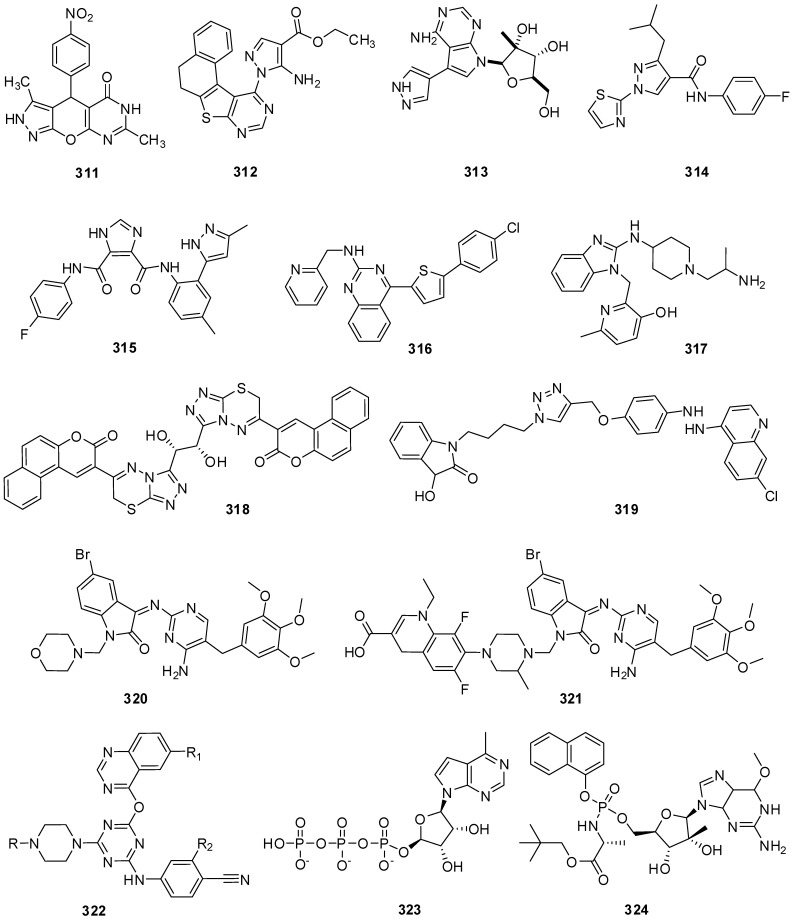
Miscellaneous *N*-heterocyclic derivatives with antiviral activity.

Several new pyrazolo[4′,3′:5,6]pyrano[2,3-d]pyrimidine analogs were evaluated for antiviral activity toward HSV type-1. The findings showed that when compared to other compounds, compound **311** has the most significant impact on HSV-1 (Figure 17). When the dose was increased from 20 to 40 g per 10^5^ cells, the antiviral activity improved from 63% to 95% [403]. The substituted pyrazole molecule **312** showed the most substantial anti-HAV potential at a dosage of 20 μg per 105 cells compared to the fused pyrazolopyrimidine derivatives, according to the structural activities’ correlations of the testing data. It demonstrates that, compared to controls, pyrazole-pyrimidine ring fusion reduces anti-HAV action (amantadine and acyclovir). 

Di Francesco et al. presented a novel class of nucleoside inhibitors of the polymerase NS5B of the hepatitis C virus (HCV) possessing pyrazole rings. The pyrazole molecule **313** (Figure 17) from the series showed a noticeably enhanced intrinsic effect by inhibiting polymerase NS5B with an NTP IC_50_ of 0.5 μM and displaying exceptional antiviral activity in the plasmid analysis with an EC_50_ value of 7.8 μM [404]. 

Jia et al. identified novel pyrazole hybrids using pharmacophore and bioisosterism hybrid methods as non-nucleoside HBV inhibitors, and their IC_50_ values were 2.22 μM and 24.33 μM, respectively; molecule **314** demonstrated intense action against the excretion of HBeAg and HBsAg [405].

In vitro*,* inhibitory effects on the yellow fever virus (YFV) and the dengue virus (DENV) were evaluated using a new category of imidazole-4,5-dicarboxylic acid (I45DC), which showed micromolar action against both viruses. The imidazole 4,5-dicarboxamide (I45DC) hybrid **315** demonstrated effective anti-dengue virus activity, as indicated according to the outcomes of an elevated screening method employing the dengue virus-2 replicon (EC_50_ = 2.5 μM). Antiviral drugs with strong inhibitory properties have been shown, including the chlorobenzyl-thiophene analog **316** (Figure 17) [343]. 

JNJ-2408068 (**317**) was 100,000 times more effective than ribavirin (EC_50_ =15 μM) at inhibiting RSV with an EC_50_ value of 0.16 nM against some lab strains. Compound **317** was also shown to inhibit the release of proinflammatory cytokines IL-6, IL-8, and Rantes from RSV-infected A549 cells [406]. As a prominent molecule, coumarin is a naturally occurring substance with extensive medical applications. In vitro antiviral activity was used to select novel 3,3′-(3,3′-(dihydroxy/hydroxyethane-1,2-diyl)bis(7*H*-[1,2,4]triazolo[3,4-*b*][1,3,4]thiadiazine-6,3-diyl))bis(2*H*-chromen-2-ones), and the docking simulation approach was used to corroborate this selection. Molecule **318** (Figure 17), one of the substances tested, showed antiviral efficacy against the H1N1 virus [407]. 

A series of 1*H*-1,2,3-triazole-tethered isatin-7-chloroquinoline and 3-hydroxy-indole-7-chloroquinoline conjugates have been synthesized and evaluated for their antimalarial activity against the chloroquine-resistant W2 strain of *Plasmodium* falciparum. The most potent of the test compounds (**319**) in Figure 17 with an optimum combination of a 3-hydroxy-indole ring and an *n*-butyl linker displayed an IC_50_ value of 69 nM [408]. 

Sriram et al. designed molecules **320** and **321** depicted in Figure 17 and they were revealed to be the most potent long-range chemotherapeutic agents that are efficient towards HCV, *M. tuberculosis*, HIV, and different harmful bacteria due to their multiple analogs, such as the lomefloxacin and ciprofloxacin moiety at the position of *N*-1 [409]. 

The anti-HIV potential of compound **322** (Figure 17) developed by Patel and colleagues was investigated towards two different HIV strains, namely HIV-2 (ROD) and HIV-1 (IIIB). In the anti-HIV test, these analogs displayed an IC_50_ value of fewer than 15 μg/mL. A hybrid with an attached quinazoline substituent to the s-triazine core and an *N*-methyl piperazine ring demonstrated a therapeutic efficacy of 5 in the anti-HIV test towards the HIV-1 (IIIB) strain [410,411,412,413].

The adenosine derivative (**323**) (Figure 17) exhibited potent antiviral activity against the RNA viruses poliovirus (PV) and dengue virus (DENV) in vitro [327]. The 2′-C-methyl modified nucleoside INX-08189 (**324**), a phosphoramidate prodrug of 6-O-methyl-2′-C methylguanosine (Figure 17), showed a nanomolar inhibition effect against DENV-2. When combined with ribavirin, INX-08189 (**324**) had a powerful synergistic anti-DENV action in vitro [414]. 

**Figure 18:**
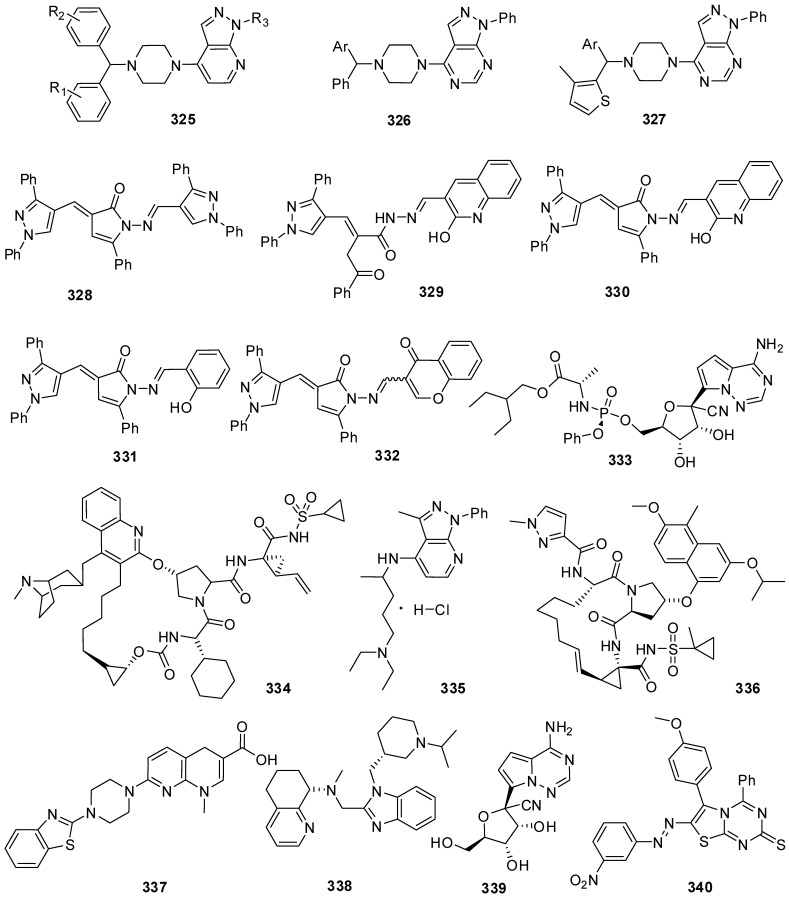
Miscellaneous *N*-heterocyclic derivatives with antiviral activity.

The antiviral effectiveness of pyrazolo[3,4-d] pyrimidines with piperazine ring systems (**325–327**) towards enterovirus was investigated. At nanomolar concentrations less than 0.7 μM, all compounds exhibited exceptional action towards Coxsackie viruses. According to the SAR, the polar diary methyl group at the piperazine and the phenyl group at the *N*-1 position significantly improved the novel analogs’ in vitro anti-enteroviral efficacy. In all instances, low MIC values of 0.37–1.25 μM for **326**, 0.32–0.65 μM for **327**, and 0.41–1.98 μM for **325** analogs (Figure 18) with therapeutic indices of >25 were found against EV71 and CVB3 pathogens [415].

Morsy et al. described the preparation and scheme of some prominent new pyrrolone hybrids represented in Figure 18 having a pyrazole moiety with the exhibition of their antiviral approach in definite pathogen-free (SPF) chicken embryos against Newcastle disease virus (NDV) and the immune-enhancing characteristics of these substances in SPF chicks. The results discovered that pyrazole hybrid **328** and quinoline hybrids **329** and **330** showed 100% resistance against NDV. In turn, the hydroxyphenyl hybrid **331** presented a 95% resistance, while chromone hybrid **332** presented a 90% resistance [68].

GS-5734 (**333**) is a phosphoramidite prodrug (Figure 18) that showed 10–40 times more antiviral potential towards TBEV serocomplex members than the genetic analog. The process by which a prodrug is transformed into a biologically active form may be responsible for the increased antiviral impact. To cure flaviviral infectious diseases, the observed SI values were 2.4–8.3 and has limited clinical efficacy [416]. (SI values stands for selectivity index values. The SI value is a measure of the selectivity of a compound for its target. It is calculated by dividing the CC_50_ value by the EC_50_ value. The EC_50_ value is the concentration of a compound that produces 50% of its maximum effect. A higher SI value indicates that a compound is more selective for its target.)

Shah et al. reported the synthesis of quinoline derivatives as HCV drug candidates to target the NS3/4a protease, which plays a crucial role in processing viral protein and replicating viral RNA. It also inhibits the production of interferons that can enhance the immune system against viral infection. The new scaffold **334** (Figure 18) was developed based on previous clinical candidate MK-5172, with a quinoline moiety instead of quinoxaline [350]. This compound exhibited improved potency against the HCV genotype A165 mutant, enhanced the interaction with the binding pocket HCV NS3 protease compared to reference MK-5172, and had a moderate pharmacokinetic profile in rats in the SAR study. Compared to MK-5172, the replacement of quinoxaline for quinoline in compound **334** enhanced its interaction with the binding pocket HCV NS3 protease.

Chloroquine is a weak base that displays direct antiviral potential against numerous viruses by inhibiting the pH-dependent steps of the replication [396,417]. Keep in mind that chloroquine is used as a reference drug for the Coxsackievirus B-3. Luiza et al. prepared and tested two new analogs as antiviral agents: N^4^-(3-methyl-1-phenyl-1*H*-pyrazolo[3,4-b]pyridine)-N^1^, N^1^-diethyl-1,4-pentane diamine (**335**). In vitro*,* the antiviral efficacy of molecule **335** against Coxsackievirus was observed (Figure 18) [418]. 

Moreau et al. approached the synthesis of macrocyclic acyl sulfonamides with a pyrazole substrate as HCV NS3 protease inhibitors with effectiveness against clinically significant resistant variants. When tested against mutant R155K 1a and D168V 1b, compound **336** (Figure 18) showed incredibly strong antiviral action with EC_50_ values of 0.14 nM and 1.0 nM, respectively [419]. 

The ability of the 1,8-naphthyridone analog to target HIV-1 tat-mediated transcription and to prevent HIV-1 replication was tested in combination with piperazine moieties. The potential antiviral effect was examined in the benzothiazole ring analog **337** (Figure 18) against anti-HIV-2 (ROD) and anti-HIV-1 (IIIB) strains in chronically infected cells with EC_50_ values of 0.03 μg/mL and 0.02 μg/mL and therapeutic index levels of **457** and **422**, respectively [420].

As CXCR4 antagonists, Miller et al. proposed a range of *N*-substituted benzimidazoles. The molecular entity such as molecule **338** (Figure 18) with the IC_50_ value of 2 nM, the 2-fold protein shift, and the 1000-fold cytotoxicity window stood out among the others as having promising antiviral potential. The availability of compounds with low nanomolar anti-HIV potential was dramatically improved through stereochemical optimization and side-chain alteration [421].

Gilead Sciences, Inc. discovered GS-441524 (**339**) (Figure 18), a 1′-cyano modified C-nucleoside, to treat filovirus illnesses and display impressive antiviral properties towards pneumo- and paramyxoviruses [422]. It exhibited YFV and DENV-2 micromolar inhibition effects, with EC_50_ values of 9.46 and 11.0 mM, respectively [423]. Surprisingly, significantly unfavorable in vitro activities (30–51.2 mM) were recorded for tick-borne flaviviruses and WNVs like OHFV, TBEV, AHFV, and KFDV [416,423]. Pande et al. have developed certain novel 6-aryl-7-arylazo-4-phenyl-2*H*-thiazolo-[3, 2-a]-1,2,5-triazine-2-thione analogs **340** (Figure 18) and analyzed their effectiveness against the virus that causes Ranikhet illness [424].

## 25. Conclusions

The domain of antiviral research has been significantly enriched by a comprehensive article that explores the fascinating field of *N*-heterocycles. This article reveals the promising antiviral properties of several five- and six-membered *N*-heterocyclic compounds along with their structure–activity relationships. From pyrazoles to nucleosides, the wide range of compounds explored in this study provides a clear picture of the potential these *N*-heterocycles possess in the field of antiviral treatments, and the wide range of molecules investigated. The inspiring exploration of pyrazoles, imidazoles, and their aromatic counterparts such as benzimidazoles and benzothiazoles, amongst others, has discovered plenty of compounds exhibiting fascinating antiviral properties. The precise correlation between molecular structure and antiviral potency is analyzed in this article via the perspective of structure–activity relationships. The findings presented not only provide the complex mechanisms underlying these compounds’ activities but also provide a roadmap for designing novel antiviral agents in the future. This article stands out for its comprehensive view, which summarizes the advancements made over the last 15 years in the synthesis and exploration of these *N*-heterocyclic molecules. In the medical world, there is hope because of the potential these chemicals demonstrate, functioning at different stages of the viral lifecycle. It is trustworthy that the compounds discussed in this article will eventually find a place in the medical arsenal because history has demonstrated that some of the most potent antiviral drugs have their roots in the field of *N*-heterocycles. Today, as viruses continue to evolve and provide a constant threat to global healthcare systems, the development of new antiviral drugs is not only a scientific endeavor but also a social necessity. The compounds under scrutiny in this article open the doors to potential breakthroughs in antiviral medicine. With continued development, these compounds have the potential to emerge as the next level of protection against viral enemies as they move from laboratory discovery to clinical applications. In conclusion, the elucidation of antiviral activities and structure–activity relationships of diverse *N*-heterocycles through this article marks a significant move in antiviral research. The knowledge gathered from this investigation is expected to have a significant impact on the scientific community as well as, possibly, on the field of medicine very soon. The light of hope for better antiviral therapies is becoming brighter because of these precisely carried out investigations.

## Data Availability

No new data were created or analyzed in this study. Data sharing is not applicable to this article.
